# GISS‐E2.1: Configurations and Climatology

**DOI:** 10.1029/2019MS002025

**Published:** 2020-08-11

**Authors:** Maxwell Kelley, Gavin A. Schmidt, Larissa S. Nazarenko, Susanne E. Bauer, Reto Ruedy, Gary L. Russell, Andrew S. Ackerman, Igor Aleinov, Michael Bauer, Rainer Bleck, Vittorio Canuto, Grégory Cesana, Ye Cheng, Thomas L. Clune, Ben I. Cook, Carlos A. Cruz, Anthony D. Del Genio, Gregory S. Elsaesser, Greg Faluvegi, Nancy Y. Kiang, Daehyun Kim, Andrew A. Lacis, Anthony Leboissetier, Allegra N. LeGrande, Ken K. Lo, John Marshall, Elaine E. Matthews, Sonali McDermid, Keren Mezuman, Ron L. Miller, Lee T. Murray, Valdar Oinas, Clara Orbe, Carlos Pérez García‐Pando, Jan P. Perlwitz, Michael J. Puma, David Rind, Anastasia Romanou, Drew T. Shindell, Shan Sun, Nick Tausnev, Kostas Tsigaridis, George Tselioudis, Ensheng Weng, Jingbo Wu, Mao‐Sung Yao

**Affiliations:** ^1^ SciSpace LLC New York NY USA; ^2^ NASA Goddard Institute for Space Studies New York NY USA; ^3^ Center for Climate Systems Research, Earth Institute Columbia University New York NY USA; ^4^ CIRES University of Colorado Boulder Boulder CO USA; ^5^ NOAA/ESRL/Global Systems Laboratory Boulder CO USA; ^6^ Goddard Space Flight Center Greenbelt MD USA; ^7^ SSAI Greenbelt MD USA; ^8^ Department of Applied Physics and Applied Mathematics Columbia University New York NY USA; ^9^ Department of Atmospheric Sciences University of Washington Seattle WA USA; ^10^ Department of Earth, Atmospheric and Planetary Sciences Massachusetts Institute of Technology Cambridge MA USA; ^11^ Department of Environmental Studies New York University New York NY USA; ^12^ Department of Earth and Environmental Sciences University of Rochester Rochester NY USA; ^13^ Barcelona Supercomputing Center Barcelona Spain; ^14^ ICREA, Catalan Institution for Research and Advanced Studies Barcelona Spain; ^15^ Climate, Aerosol, and Pollution Research, LLC Bronx NY USA; ^16^ Nicholas School of the Environment Duke University Durham NC USA

**Keywords:** General Circulation Model, climate change, CMIP6, NASA GISS

## Abstract

This paper describes the GISS‐E2.1 contribution to the Coupled Model Intercomparison Project, Phase 6 (CMIP6). This model version differs from the predecessor model (GISS‐E2) chiefly due to parameterization improvements to the atmospheric and ocean model components, while keeping atmospheric resolution the same. Model skill when compared to modern era climatologies is significantly higher than in previous versions. Additionally, updates in forcings have a material impact on the results. In particular, there have been specific improvements in representations of modes of variability (such as the Madden‐Julian Oscillation and other modes in the Pacific) and significant improvements in the simulation of the climate of the Southern Oceans, including sea ice. The effective climate sensitivity to 2 × CO_2_ is slightly higher than previously at 2.7–3.1°C (depending on version) and is a result of lower CO_2_ radiative forcing and stronger positive feedbacks.

## Key Points

- GISS‐E2.1 is an updated climate model version for use within the CMIP6 project
- Atmospheric composition is calculated consistently in all model versions
- Results demonstrate a significant improvement in skill in a climate model without changes to atmospheric resolution

## 1 Introduction

The evaluation and assessment of climate models that are being used for attribution of past change and projections of future change has, for the last two decades, been dominated by the Coupled Model Intercomparison Project (CMIP). This is an internationally organized project run by the community and with almost universal participation from climate modeling groups across the world. The latest iteration (Phase 6) started accepting data in 2018 (Eyring et al., 2016) in anticipation of the upcoming Intergovernmental Panel on Climate Change (IPCC) 6th Assessment Report (AR6) due in 2021.

Climate modeling at the Goddard Institute for Space Studies (GISS) has a long pedigree dating back to the late 1970s (Hansen et al., 1983, 2002, 1997) and has participated in almost all phases of the CMIP project, notably in CMIP3 and CMIP5 (Schmidt et al., 2006, 2014). Community experience over the last decade has demonstrated that constrained structural diversity in climate modeling is essential for elucidating important connections between processes and outcomes, and GISS models, with their distinct pedigree, have an important and continuing role to play in providing part of that diversity (Knutti et al., 2013). However, for that role to be successful, GISS needs to maintain and improve model realism (better process inclusion and higher skill) and continue participation in international and national climate model assessment projects. These projects allow model developers to benefit from the very broad scrutiny of results in these public archives from interested researchers and users across the world.

This paper is a description and an initial assessment of the GISS‐E2.1 climate model, the first GISS contribution to CMIP6. This model version was developed as part of a long‐term strategy to improve model performance as much as possible without a significant jump in computational resources, building from the GISS‐E2 models used in CMIP5. This exercise could be seen as the result of a much longer tuning process than is generally undertaken with a new model (Schmidt et al., 2017). This paper then focuses on the modern climatology in the historical simulations, namely, the satellite era from 1979. Details of the composition modeling used are in Bauer et al. (2020). The transient forcings and responses are discussed in Miller et al. (2020), and future scenarios will be discussed elsewhere. Carbon cycle enabled versions are discussed in Ito et al. (2020). A model version (E2.2) with finer layering and a higher model top is described in Rind et al. (2020), and a more substantially improved model version with better microphysics and a new cubed‐sphere grid (E3) will be described elsewhere.

The outline of this paper is as follows: In section 2, we document updates to the code and input data sets. Section 3 describes the design of the simulations discussed here, and section 4 describes the coupled model tuning. The modern climatology (including some aspects of the internal variability) of the model for the satellite period is assessed in section 5. In section 6, we briefly discuss the climate sensitivity across the configurations (though a deeper exploration is available in Miller et al., 2020). Section 7 summarizes our conclusions.

### 1.1 Nomenclature

The series of GISS ModelE versions used in this and previous CMIP iterations, have been GISS‐E‐R, GISS‐E‐H, and GISS‐AOM (in CMIP3, with the R and H denoting different ocean models Hansen et al., 2007; Schmidt et al., 2006; Sun & Bleck, 2006; and AOM referring to a different coupled model; Russell et al., 1995) followed by GISS‐E2‐R and GISS‐E2‐H in CMIP5 (Schmidt et al., 2014), and GISS‐E2.1‐G and GISS‐E2.1‐H (in CMIP6). Other CMIP6 versions include GISS‐E2.2‐G/H and GISS‐E3‐G. Some versions (denoted by ‐CC) also include an interactive carbon cycle (Romanou et al., 2014). In CMIP5, there were three formal versions of the models that varied according to the degree of interactivity in atmospheric composition (physics‐version=1,2, or 3). In CMIP6, physics‐version=2 has been dropped, physics‐version=1 denoted as NINT (for noninteractive) uses offline whole‐atmosphere ozone and aerosol fields from physics‐version=3 the OMA model as described in Bauer et al. (2020), and two new aerosol schemes have been added: TOMAS (denoted by physics‐version=4) (Lee & Adams, 2012) and MATRIX (physics‐version=5) (Bauer et al., 2008), which will be described elsewhere. For forcings, there is an additional labeling parameter f# in the CMIP6 database, which is used to denote variations of concentrations, emissions, and other input data. In the E2.1 submissions three versions have been made available for the historical runs; f1, f2, and f3, which have different composition forcings (see section 2.1.3). Documentation of these conventions in all GISS CMIP6 submissions will be maintained and updated online (https://data.giss.nasa.gov/modelE/cmip6/).

## 2 Model Code Changes

Code changes since GISS‐E2‐R/H (Schmidt et al., 2014) consist of replacement or structural variation of some parameterizations, updating of input files, bug fixes, and retuning of specific parameters. These changes have been driven by internal and external identification of unsatisfactory performance, desired improvements in physical realism in parameterizations, and updates of observational data sets used either as input or evaluation. This section lays out the reasons for the changes and the specific changes made. Notably, with the exception of additional layers in the ocean models (8 in E2.1‐G to reach 40, 6 in E2.1‐H to reach 32), no other changes were made to the horizontal or vertical resolution in any component. The atmospheric resolution is 2 × 2.5 latitude/longitude, with 40 layers in the vertical, and a model top at 0.1 hPa.

The main focus of the developments was to address unrealistic aspects in the CMIP5 simulations, notably poor Southern Ocean sea surface temperature (SST) and sea ice (a common problem across CMIP5 (Hyder et al., 2018)), excessive ocean mixing, and precipitation pattern biases which were evident in Schmidt et al. (2014). Additionally, through the intense analysis by the wider community of the CMIP5 simulations, additional issues were identified that led to subsequent bug fixes or re‐calibrations of the code (for instance, the assessment in Prather et al., 2017, led to a reexamination of the ozone chemistry, and the authors of Hezel et al. (2012) alerted us to an issue with snow cover over sea ice). Lastly, new functionality was required to accommodate more complex emission input data and irrigation effects. The specifics of the changes are outlined in the following sections.

### 2.1 Atmospheric Processes

As stated above, atmospheric resolution is the same as in the CMIP5 model, including the number of layers. However, a change was made to the manner in which terrain‐following (sigma) layers in the troposphere transition to constant‐pressure layers in the stratosphere. In E2, the transition is abrupt, occurring at 150 hPa. For E2.1, the option was activated to use a smooth transition, centered at 100 hPa with a half‐width of approximately 30 hPa. This change removes some artifacts previously seen in the diagnostics but negatively impacted the stratosphere circulation slightly.

#### 2.1.1 Radiative Transfer

The total solar irradiance has been updated based on new satellite calibrations (Kopp & Lean, 2011) to have a base value of 1,361 W m^−2^ (compared to 1,366 W m^−2^ in GISS‐E2) though this is not expected to have any impact on the climatology or sensitivity once the models have been returned for radiative balance (Rind et al., 2014). Spectral irradiance values have also been updated to the latest estimates (Coddington et al., 2016).

Further calibration of the GISS‐E2 radiation framework against line‐by‐line results led to a few improvements for E2.1. Most notably, noncontinuum absorption of shortwave radiation by water vapor was significantly increased, thereby rectifying a problem subsequently highlighted in analyses of the CMIP5 ensemble (DeAngelis et al., 2015). In the longwave region, a systematic increase of Outgoing Longwave Radiation (OLR) of a few W m^−2^ was the main outcome of optimizations of lookup tables for finer model layering and larger training sets of atmospheric profiles. The HITRAN 2012 spectroscopy (Rothman et al., 2013) was also incorporated, though with negligible impact. The improvements to clear‐sky SW and LW skill relative to E2 and other schemes can be seen in the intercomparison of Pincus et al. (2015).

A small but consequential error in the snow masking of vegetation (where a constant snow density was used instead of the computed predicted snow density) was fixed, thereby reducing the area fraction of old, compacted snow and hastening springtime snowmelt.

A number of small additional changes were made to the inputs to the radiative transfer code: (1) We increased the longwave optical depth for dust by 30% to account for the longwave scattering effect (which was not included in E2) (Schmidt et al., 2006). (2) The lensing effect of sulfate and nitrate coatings on BC was parameterized by increasing the shortwave optical depth for BC by 50%. And (3) an improved distinction between ozone and total odd oxygen was made (which causes the upper stratosphere to cool slightly).

#### 2.1.2 Clouds, Convection, and Boundary Layer

As described in Kim et al. (2012), Del Genio et al. (2012), and Del Genio et al. (2015), modifications to the cumulus parameterization in GISS‐E2 led to a more realistic amplitude of variability associated with the Madden‐Julian Oscillation (MJO) in GISS‐E2.1. GISS‐E2.1 retains the basic entraining double plume updraft‐downdraft framework used in GISS‐E2, but with the following changes: (1) The entrainment rate coefficient of the more weakly entraining plume is increased from 0.3 to 0.4, thus increasing the sensitivity of convection to environmental humidity. (2) The partitioning between convective precipitation that descends and has the potential to evaporate in the environment rather than in the downdraft is increased from 0% to 50%, thus increasing the sensitivity of humidity to convection. (3) Downdraft buoyancy, which was determined solely by temperature in GISS‐E2, is now based on virtual temperature including condensate loading. (4) A previous limit on the cumulus mass flux that inadvertently resulted in zero entrainment rates at high altitudes in strongly convecting environments was eliminated.

**Figure 1:**
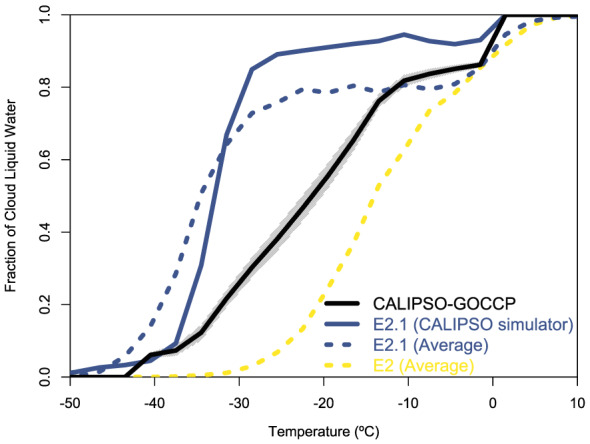
Cloud liquid fraction as a function of local temperature. The black solid line presents CALIPSO‐GOCCP observations over 2007–2016 (shading is the 95% range in the standard error of the annual mean) (Cesana, Chepfer, et al., 2016). E2 and E2.1 results are over 2007–2015. The CALIPSO simulator (Cesana & Chepfer, 2013) applied to E2.1 is the solid blue line, and the liquid mass fraction computed from monthly average condensate amounts is shown for E2.1 (blue dashed) and E2 (yellow dashed). Nonzero E2.1 liquid mass fraction at temperatures colder than −35°C is due to the use of monthly averages.

The most impactful E2.1 update to the stratiform cloud parameterization concerns the treatment of glaciation in the mixed‐phase temperature range. In E2, glaciation in a given grid cell was a probabilistically timed event after which no supercooled liquid can exist or form until all ice has disappeared and the phase decision can “reset” for a new cloud. Within the single‐phase cloud condensate framework inherited from E2, E2.1 attempts to model glaciation in a more continuous manner via a temperature‐dependent autoconversion rate of supercooled liquid to ice precipitation. This rate is rapid at the homogeneous freezing temperature of −35°C and decreases linearly toward the warm‐cloud autoconversion rate at −5°C. Relative to the new cloud reset mechanism in E2, this “virtual” mixed‐phase representation significantly increases the amount of supercooled water cloud in the Southern Ocean and the Arctic in E2.1. The increase in supercooled water amount was partially counteracted for initial tuning purposes by multiplying the effective radius for optical depth calculations by 1.1, rather than by increasing liquid autoconversion rates. While the lack of true mixed‐phase microphysics in E2.1 constrains the ice component to be merely diagnostic in any evaluation of phase partitioning for tuning purposes, the retrospective evaluation in Figure 1 suggests that availability and consideration of this target would have led to an upward tuning of liquid autoconversion rates at temperatures colder than −15°C.

The regime‐specific threshold relative humidity for stratiform cloud formation in E2 was dependent upon moist convective activity, resolved vertical motion, and altitude (near the surface). Convective area also restricted the maximum coverage of stratiform cloud. The E2.1 code was modified as follows: (1) the coverage restriction is no longer applied above convective cloud top, (2) the dependence on vertical motion was dropped, since its application criterion did not distinguish fronts from other structures, and (3) altitude is taken to be relative to local planetary boundary layer (PBL) height rather than a fixed 850 hPa, better demarcating cloud‐topped boundary layers from the free troposphere (where threshold relative humidity is *U*_*a*_). As in E2, *U*_*a*_ is the primary vehicle for the TOA radiation balancing process described in section 4; here we note that the updates described in this section collectively produce a moister and brighter atmosphere, thus requiring a compensating increase of *U*_*a*_ to maintain top‐of‐the‐atmosphere radiative balance.

The modifications of the turbulence parameterization within and above the PBL (Yao & Cheng, 2012) from GISS‐E2 include (1) the nonlocal vertical transport scheme for virtual potential temperature, specific humidity, and other scalars is updated from the (Holtslag & Moeng, 1991) scheme to the more robust Holtslag and Boville (1993) scheme; (2) employing the turbulence length scale formulation obtained from the large eddy simulation data by Nakanishi (2001); (3) using the more realistic “Richardson number criterion” rather than the “TKE criterion” to calculate the PBL height, following Troen and Mahrt (1986) and Holtslag and Boville (1993); and (4) modifying the similarity law near the surface in extreme stability conditions (Zeng et al., 1998). With the above modifications, the relative humidity and low cloud cover have better vertical structures due to greater transport of water vapor in the PBL. The differences in the diagnosed PBL height between the E2.1 and E2 versions correlate well with the differences in the total cloud distribution over oceans. This newer parameterization leads to improvement in cloud and radiation fields in the extratropics (see section 5.2 below). Tropical low clouds were not specifically targeted, as they require finer layering at low levels and a cloud‐enabled PBL scheme, which will be demonstrated in the documentation for the E3 version.

#### 2.1.3 Composition and Chemistry

The basic NINT simulations that are the focus of this paper do not have interactive composition, but the background fields of ozone and aerosol concentrations are derived from simulations of the interactive OMA version of the model, run under AMIP conditions (Bauer et al., 2020). Thus, the numerous, relatively minor updates and improvements to the composition modules affected these runs and so are described here for completeness.

All anthropogenic and biomass burning emissions of short‐lived species were updated to CMIP6 specifications (Hoesly et al., 2018; van Marle et al., 2017) and are now prescribed annually, rather than by decadal interpolation as in CMIP5. Coding changes include (1) calculating solar input to photolysis code using higher wavelength resolution; (2) updating the photolysis calculations to use up to three sets of temperature‐dependent cross sections rather than 2; (3) harmonizing the heterogeneous chemistry reaction rate calculations in the stratosphere to use the identical aerosol surface areas as those in the radiation code (typically satellite‐derived extinction values); (4) updating reaction rate coefficients from the JPL 2000 to the 2011 compendium (Sander et al., 2011); (5) removing an imposed minimum tracer value that had led to large mixing ratios in high latitude grid boxes at high altitudes where total air masses are small; (6) expanding the representation of reactions including atomic hydrogen (no longer limited to specific pressure ranges); (7) expanding aircraft emissions to include more species; (8) correcting the amount of ozone input in photolysis calculations to use the grid box top rather than the mid–grid box value, which led to ozone chemistry biases (Prather et al., 2017). The harmonization of aerosol surface areas in (3) identified a coding error that led to large underestimates in volcanic aerosol surface areas for chemistry in the stratosphere. The two sets of runs denoted by f1 and f2 forcings reflect the impacts of that change.

We also include simulations with a third set of forcings f3 that use the ozone and aerosol composition from the high‐top E2.2 (OMA) simulations (Rind et al., 2020). These simulations have a more realistic stratospheric circulation and age of air and improved stratosphere‐troposphere exchange, though they use a differently tuned convection parameterization. Small adjustments in the photolysis tuning to correct for circulation‐induced biases in high latitude NO_*x*_ and O_3_ were removed as well. The ozone field is improved in the tropics related to reduction in the Brewer‐Dobson circulation strength and weaker transport of ozone‐rich air to high latitudes, with some improvements in tropical lower stratospheric temperatures. The impact of these changes is also seen in a different response in ozone to volcanic eruptions.

Several updates were made to lightning NO_*x*_ production in the chemistry module. The default flash rate parameterization remains a function of convective cloud depth, separately determined over land and sea (Price & Rind, 1994). However, the calculation is now done using altitude above ground level rather than sea level, eliminating spurious lightning over high‐altitude regions such as Antarctica. The land and marine flash rate equations are separately tuned to reproduce the respective present‐day mean values from the Lightning Imaging Sensor (LIS) and Optical Transient Detector (OTD) satellite climatology (Cecil et al., 2014). Flash rates are converted to column NO_*x*_ production rates using a fixed NO_*x*_ yield per flash assumption. These are then distributed vertically from the surface to the local cloud top height using the unimodal probability distribution functions of Ott et al. (2010) instead of the earlier bimodal distribution of Pickering et al. (1998). The NO_*x*_ yield per flash is determined such the model reproduces the present‐day methane chemical lifetime of 9.7 yr (Prather et al., 2012). This results in 290 mol N per flash, yielding a global mean of 6.4 Tg N yr^−1^. This is slightly lower than in E2 (7.3 Tg N yr^−1^) (Shindell et al., 2013a) and falls within the relatively large range of estimates for the present‐day lightning NO_*x*_ source (2–8 Tg N yr^−1^) (Murray, 2016).

The E2.1 version of the aerosol module OMA is documented by Bauer et al. (2020), who evaluate its performance (for CMIP6 forcings) against satellite, surface network, and ice core data. Unchanged in structure from E2, in which it was named TCADI, the species treated by this module are dust, sea‐salt, sulfate, nitrate, ammonium, and carbonaceous aerosol (black and organic carbon, including the NO_*x*_‐dependent formation of SOA and methanesulfonic acid formation). The following updates were made: (1) increased in‐cloud ammonia dissolution to account for dissociation, thereby remedying the overabundance of nitrate aerosol in E2 (Mezuman et al., 2016; Nazarenko et al., 2017); (2) tuning of the parameterized *e*‐folding time for hydrophobic to hydrophilic BC conversion (a proxy for aging lifetime) to match that of MATRIX (Bauer et al., 2008), which does include physically based aging calculations as part of the aerosol microphysics. The new aging timescale for OMA was evaluated using ice cores and HIPPO flight campaign data in Bauer et al. (2013); (3) updates to the dust representation as discussed below.

We updated the heterogeneous chemistry calculations for the formation of nitrate and sulfate coatings on the surface of soil dust particles by uptake of nitric acid and sulfur dioxide, respectively, which were originally described by Bauer et al. (2004) and Bauer and Koch (2005). Dust properties are now retrieved from the dust module, instead of being defined separately in the heterogeneous chemistry module, to make those properties consistent with the rest of the model. This concerns the boundaries of the six dust bins (0.1–0.2, 0.2–0.5, 0.5–1, 1–2, 2–4, and 4–8 μm particle diameter), which are used for coatings on dust particles, the dust particle densities, and the weights that are used to partition the total clay which is advected as a bulk species in the model. The weights reflect the size distribution of dust, compared to the previous version where inadvertently only the largest clay bin was considered. An erroneous calculation of the dust number concentration, which led to an overestimate, was also corrected. The net effect of the changes is to reduce masses of sulfate and nitrate coating on dust by an order of magnitude due to lower uptake of the precursor gases sulfur dioxide and nitric acid, respectively. The global precursor masses in the atmosphere are larger by about 6% and 9%, respectively, with significantly larger increases over North Africa, Middle East, and Central Asia, where dust concentration is elevated. In turn, particulate nitrate aerosol mass is up to five times higher over equatorial Africa and India and sulfate aerosol is up to 50% more abundant in the northern hemisphere.

The default dust aerosol tracers in the OMA version follow the approach of Cakmur et al. (2006), with the difference that the emitted silt and clay fractions of total dust and the emitted total dust mass are optimized in two successive steps, instead of simultaneously. The two‐step approach reduces the emitted relative fraction of clay‐dust mass (now about 8% of all dust mass over the size range 0.1–32 μm for OMA), thus making the model better agree with recently published research on the global size distribution of dust in the atmosphere (Kok et al., 2017).

**Figure 2:**
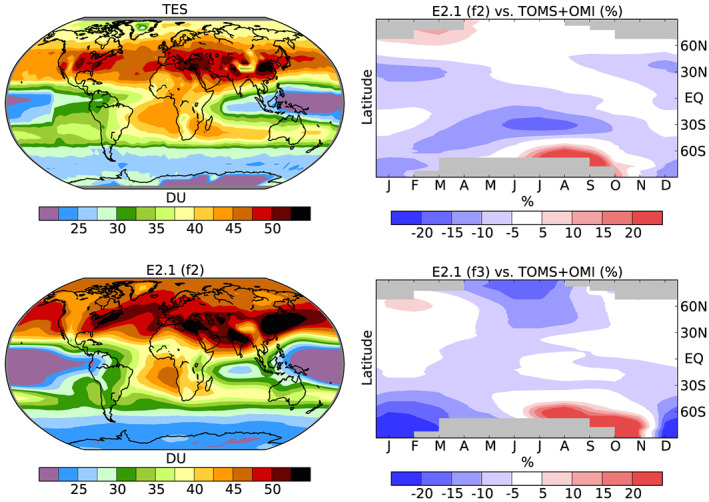
Left column: Annual average 2005–2009 tropospheric column ozone (DU) in TES observations (top) and in E2.1 f2 (bottom). The tropopause is defined using the NCEP 2005–2009 monthly values for TES and the model's internally calculated values for E2.1. Right column: 2000–2010 average of zonal mean, seasonal total column ozone (DU) as a percent difference with respect to TOMS/OMI observations for the same years for E2.1 f2 (top) and E2.1 f3 (bottom).

Ozone distributions used in the NINT models are generally similar to those in prior versions. Changes to chemistry have resulted in modest improvements to comparisons with observational data in the troposphere (Table 1). For example, the average bias near the surface (900 hPa) has been reduced from 6.6 (22%) in E2 (Shindell et al., 2013b) to 3.6 (12%) in E2.1 (f2). Modeled polar ozone in this configuration is biased as the Brewer‐Dobson circulation to high latitudes is too strong in winter, leading to ozone and temperature overestimates during that season. This creates large positive biases in the lowermost stratosphere and upper troposphere from June through September over 60–90°S and smaller, but again positive, biases from January through April over 60–90°N (Figure 2). These positive winter high latitude biases are reduced in the model version used to create f3 forcing (especially in the Northern Hemisphere), with its more realistic stratospheric circulation, but that version has larger negative summertime biases in both polar regions.

**Table 1:** Ozone Differences and Biases (ppbv) Between Model E2.1‐G f2 and OMA Versions and Sonde Climatologies

Pressure level	Avg. diff.	Avg. diff.	Avg. bias	Avg. bias	Std. dev. of
(hPa)	AMIP	coupled	AMIP	coupled	observations
125	43.5	65.8	9.9	45.1	92.9
200	21.7	27.3	1.2	7.7	52.2
300	13.4	15.2	7.0	8.3	25.6
500	9.6	10.9	6.2	7.5	11.7
900	8.0	8.8	3.6	4.5	8.9

*Note*. Sonde data primarily from the 1990s and early 2000s (Logan, 1999; Thompson et al., 2007); model from 1999–2003 averages.

Comparison of the tropospheric column ozone with observations from the Tropospheric Emission Spectrometer (TES) show that the model captures many features of the distribution (Figure 2). The wintertime positive biases in the lower stratosphere are clearly visible in model overestimates of tropospheric column poleward of 50°N and 70°S. Such comparisons are highly sensitive to the tropopause definition (Shindell et al., 2013b), which is in turn sensitive to stratospheric temperature biases and so typically any widespread ozone biases seen here reflect only small differences in the altitude of the tropopause relative to observations. The model captures the maximum over the Atlantic off the west coast of Africa and the minima over the equatorial Pacific and Indian Oceans. As in E2, the minimum over the eastern tropical Pacific is too low, however, and this is likely to again dominate biases in long wave radiative fluxes due to ozone (Bowman et al., 2013). The distribution of column ozone is well represented over most of the NH midlatitudes, though the magnitude is roughly 2–4 DU too large. The global area‐weighted column average in the model is 35.4 DU for the f2 case and 34.4 DU for the f3 case, both very similar to the 35.9 DU from the TES observations (Bowman et al., 2013). Spatial correlations are broadly similar to those in E2, with an R^2^ correlation against TES of 0.86 for f2 and 0.83 for f3 (compared to 0.85 in E2) and a value of 0.68 for f2 and 0.74 for f3 against the tropospheric column estimate obtained from OMI minus MLS observations (compared to 0.71 for E2).

The other primary oxidant in the troposphere in addition to ozone is the hydroxyl radical (OH). To examine its abundance, we evaluated the residence time of methane as a proxy for OH since oxidation by hydroxyl is the main removal mechanism for methane. The residence time in E2.1 is 8.3–9.1 yr, in excellent agreement with estimates based on observations that yield a value of 9.1 ± 0.9 yr (Prather et al., 2012), indicating that tropospheric oxidation capacity due to OH is well represented.

Overall performance of the composition diagnostics is fairly similar to E2, based on comparison with the trace gas observations made in Shindell et al. (2013b). A detailed analysis suggests that over the United States and China, the model is slightly high biased in terms of the simulated tropospheric ozone column relative to TES measurements (Figure 2) and substantially low biased in terms of aerosol optical depth relative to MISR observations (Seltzer et al., 2017). The ozone biases are large enough that analyses of surface ozone impacts, such as the nonlinear effect on human health of exposure over a given threshold, would be substantially overestimated without adjusting for this bias, as is common using surface ozone from chemical transport models (Seltzer et al., 2018; Shindell et al., 2018). The ozone‐related biases in radiative forcing and hence climate are likely to be small, however, as ozone is only modestly too large and the bias appears to be systematic over time. Errors in aerosol distribution are still important and may impact the radiative trends over recent decades (Bauer et al., 2020).

As part of the comparison to E2, we note that E2 used a temperature threshold for the formation of polar stratospheric clouds (and hence the heterogeneous chemistry associated with them) (Shindell et al., 2013b), which was tuned to correct the polar ozone hole timing, despite potential biases in polar vortex temperatures. However, this was not used in E2.1. This model does, however, maintain prior practice of tuning photolysis rates at short wavelengths (<200 nm) for N_2_O and O_2_ that corrects for problems in stratospheric circulation that otherwise lead to biases in high latitude concentrations of NO_*x*_ and O_3_.

#### 2.1.4 Gravity Wave Drag

E2.1 includes orographic and frontal sources of parameterized gravity waves as in E2. Systematic reoptimization of the scheme was not performed, but two corrections required recalibration of tuning factors: (1) saturation momentum flux was reduced by a factor of approximately 2 as a result of correcting its definition (2) the metric for the presence of fronts (deformation at 700 hPa) was corrected, increasing its magnitude. The orographic wave coefficient was thus reduced (from 0.2 to 0.1) and the threshold deformation magnitude for generation of frontal waves was increased (from 0.000045 to 0.000055) and its coefficient increased from 1.5 to 1.6. Sensitivity experiments have shown that inclusion of parameterized convective gravity waves does little to improve the Middle Atmosphere circulation in this relatively low top model, unlike the orographic and frontal sources, though they are active in the E2.2 configurations (Rind et al., 2020).

### 2.2 Ocean Processes

We used two ocean model versions with E2.1, which are denoted E2.1‐G (coupling to the GISS Ocean v1 (GO1)), and E2.1‐H (coupling to HYCOM). This experimental design (as in CMIP5) was used in order isolate emergent behavior that is dependent on ocean‐atmospheric coupling and suggest where structural uncertainty in the design of the ocean module might be important. This section describes the updates in each since CMIP5.

#### 2.2.1 GISS Ocean v1

For gross ocean structure and transport metrics, the most impactful updates to E2.1‐G are in the parameterizations of mesoscale eddies and vertical mixing. In addition, a high‐order advection scheme (Prather, 1986) and finer upper‐ocean layering (an increase from 32 total layers to 40) sharpened the representation of frontal and thermocline structures in regions of weak parameterized mixing. The updates outlined here will be described more completely elsewhere, as part of parameter sensitivity studies.

A fundamental update to mesoscale eddy transport was the correction of an error in the definition of neutral surfaces in E2‐R, which drastically reduced the restratification effect. Through the lens of ocean‐only simulations and intermodel comparisons of temperature/salinity drifts and circulation metrics such as AMOC and ACC strength, subsequent work explored the consequences of controlled variations in the magnitude and structure of the mesoscale eddy diffusivity (Marshall et al., 2017; Romanou et al., 2017). Those efforts informed the creation of a moderate‐complexity 3‐D mesoscale diffusivity for E2.1‐G whose primary differences from the E2‐R scheme are (1) surface‐intensified eddies, in the form of an exponential decay of diffusivity with depth, where the location‐dependent decay scale is equal to [|*ρ*_*h*_*z*|]/[|*ρ*_*h*_|], [] denotes vertical averaging, and *ρ*_*h*_ is the horizontal gradient of potential density; (2) replacement of Rossby radius by a geographically constant nominal length scale *L* = 39 km in the baroclinicity scaling of diffusivity retained from E2‐R: *L*^2^[|*Ns*|]_1*k*_, where *N* is the Brunt‐Vaisala frequency, *s* the slope of isopycnal surfaces, and []_1*k*_ denotes vertical averaging over the upper 1,000 m depth; (3) qualitative representation of the Coriolis element in the discarded Rossby radius by a factor 1/max(.05,sin(|latitude|)) multiplying the diffusivity. The location dependence in (1) permits eddies to restratify the Southern Ocean over a large depth range, consistent with observed density structure there, while not overacting in other regions of the World Ocean (such as the North Atlantic, where the aforementioned ocean‐only experiments indicated that deep mesoscale effects can suppress the AMOC). Simplifications (2) and (3) preserve the large‐scale structure of the diffusivity distribution and its interactivity while eliminating unconstrained small‐scale structure. E2.1‐G also adopts a new representation of mesoscale transport expressed in local quasi‐isopycnal layering, circumventing some of the difficulties associated with the skew‐flux representation that was employed in E2‐R.

The E2.1‐G vertical diffusivity now includes a contribution from tidal dissipation. AMOC sensitivity to this effect is exploited as a (model‐specific) constraint on the considerable uncertainties surrounding this process. Exploratory coupled simulations, lacking the stabilizing effects of relaxation toward climatological surface salinity and a prescribed atmospheric state, systematically developed a runaway haline stratification at high northern latitudes that was the proximate cause of a weak AMOC and excessive northern hemisphere sea ice. The sole parameterization change in any atmosphere or ocean component found able to sustain a strong AMOC was tidally driven mixing, which occurs in the shallow waters bordering the North Atlantic using the dissipation distribution generated by Jayne (2009).

Ventilation of marginal seas through their connecting straits has been increased via two mechanisms in E2.1‐G, reducing salinity biases there. For straits deep enough that density contrasts can drive strong opposing flows at the surface and depth, the finer upper‐ocean layering in E2.1‐G resolves this structure, in conjunction with a slight tuning of strait depths. Second, horizontal diffusivity was increased in straits that are shallow or have weaker density contrasts. The first mechanism impacted the Red and Black seas, and the second the Baltic and Hudson. The first is the sole ventilation mechanism for straits narrower than the nominal resolution, which are parameterized using the Russell et al. (1995) one‐dimensional channel scheme that lacks horizontal mixing.

#### 2.2.2 HYCOM

HYCOM is a hybrid‐isopycnal ocean model that was used with previous coupled ModelE versions (Romanou et al., 2013; Sun & Bleck, 2006). E2.1‐H increases the number of vertical layers to 32 from 26 in E2‐H, and no longer uses a refined equatorial mesh as did E2‐H (since it no longer provided a demonstrable increase in skill in surface fields). HYCOM has traditionally used *σ*_2_ as its vertical coordinate: potential density referenced to a pressure nominally corresponding to 2 km depth. At pressures far from this reference, stable in situ stratification may be misdiagnosed as unstable according to potential density, impacting the layering scheme and vertical mixing. To ensure a monotonic potential density profile in the upper ocean under conditions of stable in situ stratification there, E2.1‐H employs *σ*_1_ (potential density referenced to 1 km). This change eliminated spurious deep convection in the Southern Ocean which inhibited formation of the summer halocline and limited sea ice extent. The resulting degradation of the abyssal diagnosis of stratification was found to be benign.

The virtual salt flux formulation of surface freshwater fluxes, employed by HYCOM for consistency with its barotropic/baroclinic mode‐splitting scheme, was corrected to conserve global salt, thereby eliminating a net source that resulted in significant positive biases in E2‐H salinity. Other fixes to ocean‐atmosphere‐ice flux coupling include (1) interpolation between grids, (2) elimination of slight inaccuracies in the sea ice mass and heat fluxes, and (3) a modification to the land topography along the coastline to reduce flux biases in atmospheric grid boxes with average land heights significantly above sea level.

### 2.3 Cryosphere

Common to both ocean models as in E2, the sea ice component of E2.1 retains the overall framework of E2, excepting the treatment of salt as a material constituent. Algorithmic changes within the framework made the most direct contributions to differences with E2 climatology and include (1) correction of an inadvertent snow‐to‐ice transformation during vertical regridding, thereby increasing snow thickness and surface albedo; (2) removal of a 10% floor on lead fraction for conditions typical of the Antarctic winter; (3) closure of leads for thick‐ice conditions typical in the Arctic, thereby reducing wintertime heat flux and ice growth there; and (4) independent horizontal advection of snow mass. Thermodynamics now follows the “Brine Pocket” (BP) parameterization (Bitz & Lipscomb, 1999; Schmidt et al., 2004), and thus, salt plays a more active role in E2.1 sea ice, affecting its specific heat and melt rates. Processes relevant to the salt budget (e.g., gravity drainage and flushing of meltwater) are consistently treated with the BP physics. The switch from the previous “Saline Ice” thermodynamics in E2 to the BP one in E2.1 led to a slight increase in multiyear sea ice thickness and of sea ice area in the Arctic, a slight reduction of the Antarctic sea ice area as well as a more physically realistic vertical profile of the salinity in the ice. Note that, as in previous studies, the overall changes in sea ice climatology especially in the Southern Oceans are driven predominantly by changes in ocean circulation and mixing (e.g., Liu et al., 2003).

### 2.4 Land Surface Processes

#### 2.4.1 Irrigation and Groundwater

While transient historical changes in irrigation was implemented as a forcing in E2 (Cook et al., 2011, 2014; Krakauer et al., 2016; Puma & Cook, 2010; Shukla et al., 2014), it was not included in the standard CMIP5 submissions. In E2.1, irrigation is now a standard component. Water demand for irrigation is calculated as described by Wada et al. (2014) using irrigation areal extent from Siebert et al. (2015) as an input. The water is drawn first from the local surface water system (including rivers and lakes), and if that is insufficient, it is assumed to be drawn from an external groundwater source (which is tracked diagnostically). Groundwater is assumed to have the same temperature as the soil and has default tracer values. Groundwater recharge is not accounted for, and so there is a small increase in total water mass (and eventually, sea level) associated with the net global groundwater draw in these simulations. These effects have a complex impact on freshwater delivery to the oceans (and hence sea level). Irrigation from local surface water sources leads to increased soil moisture and reduced river outflow, but this is dominated by net additions of groundwater which add freshwater to the climate system, about 0.2 mm yr^−1^ of global sea level equivalent in 2010 (Miller et al., 2020).

#### 2.4.2 Vegetation

As in E2, all vegetation properties affecting physical climate, with the exception of canopy conductance, are prescribed in the simulations described here, whose primary update was the incorporation of satellite‐derived distributions of vegetation characteristics, as described below. Like E2, E2.1 sees vegetation properties via the Ent Terrestrial Biosphere Model (Ent TBM), a demographic dynamic global vegetation model (DGVM) whose functionalities are gradually being coupled to ModelE (Kiang, 2012; Kim et al., 2015), including carbon cycle interactivity (Ito et al., 2020). Prescribed interannual variation of vegetation is limited to land use and land cover (LULC) change, by which historical crop and pasture cover is used to rescale the natural vegetation cover fractions in a grid cell (Ito et al., 2020; Miller et al., 2020).

We have updated the vegetation structure (including prescriptions of vegetation cover, type, height, and leaf area index) as part of ongoing Ent TBM development for E2.1, replacing E2 prescriptions based on Matthews (1983). Ent GVSD satellite data sources include land cover types and monthly varying LAI from the Moderate Resolution Imaging Spectroradiometer (MODIS) (Gao et al., 2008; Myneni et al., 2002; Tian et al., 2002a, 2002b; Yang et al., 2006), and tree heights from Simard et al. (2011), who utilized 2005 data from the Geoscience Laser Altimeter System (GLAS) aboard the ICESat (Ice, Cloud, and land Elevation Satellite). Specific leaf area (carbon mass per leaf area) data from the TRY database of leaf traits (Kattge et al., 2011) was classified for the Ent TBM 13 plant functional types (PFTs). These observed spatial distributions and leaf trait parameters together allow equilibrium behavior in plant‐atmosphere carbon exchange and internal plant carbon balances for late 20th century to early 21st century climate. The water stress algorithm, which controls the availability of soil water for transpiration, was replaced in E2.1 with a more commonly used soil water deficit‐based one (Porporato et al., 2001; Rodriguez‐Iturbe, 2000), with the goal of improving transpiration, by distinguishing soil moisture levels at which onset of water stress happens for different plant functional types.

The overall effect of these updates upon surface albedo was significant in some regions, though the overall impact upon physical climate modest compared to other components. Ent PFTs are mapped to the E2 vegetation types for radiative purposes in E2.1; reclassification of cover types directly increased the surface albedo of Australia and eastern South America by several percent. High northern latitudes became brighter via increased snow masking, though this effect was compensated by the masking correction described in section 2.1.1. Canopy conductances generally decreased using the new LAIs.

## 3 Simulation Design and Configurations

The GISS models are designed so that any experiment can be run with an appropriate level of interactivity and complexity—some experiments require the aerosol and chemistry fields to respond to and influence the surface climate, while other simulations focus on one‐way impacts. In earlier iterations, NINT historical simulations relied on calculated concentrations of aerosols and tropospheric ozone from a prior generation of models. For instance, the NINT simulations in CMIP5 (using GISS‐E2‐R or GISS‐E2‐H) used fields from Koch et al. (2011), which were calculated using the CMIP3 model (GISS‐E). In CMIP3, the aerosol and ozone fields were from the SI2000 version of the model (Koch, 2001; Koch et al., 1999) and thus were not consistent with the composition changes generated in the same‐generation interactive models (OMA or MATRIX aerosol microphysical versions) or the specified emission paths. Additionally, many key interactions present in the (computationally expensive) interactive runs (such as ozone responses to volcanoes or solar activity changes) were not represented in the CMIP5 NINT runs.

For CMIP6 we have striven for an increased coherence between forcings and model physics. Namely, we have generated all the historical composition fields for NINT versions using an ensemble of AMIP‐style runs (1860–2014) with the interactive OMA version and annually resolved CMIP6 emissions (Bauer et al., 2020). The time needed to generate new composition fields slows down production, but the resulting NINT simulations have more fidelity to the real world and reflect more processes, while being 3–4 times faster to run when compared to interactive composition versions.

### 3.1 Preindustrial Boundary Conditions

There are a few notable changes from CMIP5 for “preindustrial” (PI) conditions, which is a slight misnomer, since conditions around 1850 cannot be considered to be unaffected by industrialization, agriculture and fossil fuel use (through the background greenhouse gas levels), and explicit background levels of land use and land cover change, including irrigation (Hawkins et al., 2017). We now include a background level of irrigation along with background levels of LULC alterations and anthropogenic aerosols (see prior sections for details of the data sets used). The emissions from biomass burning are taken from the standard CMIP6 specifications but include an (uncertain) anthropogenic component. The spin‐up under PI conditions is always greater than 500 yr and drifts in global mean surface air temperature and ocean heat content are less than 0.03°C per century and 0.1 W m^−2^, respectively. This procedure does not include pre‐1850 transient changes that might be expected to still have been responsible for ocean heat content anomalies at that time (Gregory, 2010; Stenchikov et al., 2009). Nonetheless, the difference in subsurface ocean conditions from reality in 1850 are significantly larger than the impact of prior transient volcanic effects (compared to a suitable averaged background level). Experience from simulations of the last millennium in CMIP5 suggests that the differences in twentieth century transient climate resulting from this choice are minimal.

### 3.2 Historical Transients

As mentioned above, radiatively active atmospheric composition (ozone and aerosols) is taken from AMIP experiments using CMIP6‐prescribed annual emissions of aerosols, their precursors and other short‐lived reactive chemical species in E2.1 (OMA). Well‐mixed greenhouse gases, solar activity changes (affecting TSI and the spectral irradiance), and LULC (including irrigation) were specified using a mix of approaches (Miller et al., 2020). Volcanic aerosols were prescribed using precomputed aerosol depth and effective particle radius (Thomason et al., 2018), though we will also be using interactive emission‐driven volcanic effects in some future CMIP6 simulations (LeGrande et al., 2016).

It is important to note that there is substantial uncertainty in some of these drivers over time, especially in the aerosols, solar activity, and early big volcanic eruptions. We therefore plan to incorporate this uncertainty in the CMIP6 historical simulations using the f number in the ripf designation of each individual run in the CMIP6 archive.

## 4 Model Tuning

Model tuning for E2.1 loosely followed the procedure described in Schmidt et al. (2017). The first round of such optimizations is typically process oriented and does not specifically target global radiative balance, for example, tuning of convective entrainment was used to enhance MJO variability (Del Genio et al., 2015). Impactful parameters that did not participate in the first round of tuning are then potentially recalibrated to maximize agreement with their target metrics; the E2 settings for a critical relative humidity and the critical ice mass for condensate conversion (Schmidt et al., 2014) were found to remain optimal for E2.1 (*U*_*b*_=1 and WMU_*i*_=2). The following round imposes exact radiative balance for preindustrial (1850) conditions in atmosphere‐only mode, by varying the critical relative humidity *U*_*a*_. This parameter was increased from 0.54 in E2 (NINT) to 0.655 in E2.1 (NINT). Since OMA climatology differs slightly from NINT, *U*_*a*_ does as well (0.55 in E2, and 0.625 in E2.1). For E2.1 (NINT), a final round of tuning sets the aerosol indirect effect to have a global mean of −1 W m^−2^ in 2000 as it was in the CMIP5 simulations (Miller et al., 2014), following Hansen et al. (2005).

Composition tuning is also carried out in atmosphere‐only mode, and most details are described in section 2.1.3. Here we note that all such simulations include full chemistry, aerosol, and indirect effect schemes and that the indirect effect is not tuned in E2.1 (OMA). Furthermore, since some processes are extremely sensitive to small changes in climate (e.g., dust emission), some degree of iteration is required to jointly tune for their targets along with radiative balance. Finally, there is some interplay while tuning the NINT and OMA configurations, in that the latter provides composition fields used by the former, and first‐round tuning of cloud schemes is performed in the former.

Upon coupling the ocean and atmosphere models, there is an initial drift to a quasi‐stable equilibrium, which is judged on overall terms for realism, including the overall skill in the climatological metrics for zonal mean temperature, surface temperatures, sea level pressure, short and long wave radiation fluxes, precipitation, lower stratospheric water vapor, and seasonal sea ice extent. For the configuration to be acceptable, drifts have to be relatively small and quasi‐stable behavior of the North Atlantic meridional circulation and other ocean metrics, including the Antarctic Circumpolar Current, are required. El Niño–Southern Oscillation (ENSO)‐related metrics are also monitored, but they were not specifically tuned for, since the underlying tropical Pacific SST climatology was not considered to be a feasible tuning target using E2.1 vertical resolution, cloud, and boundary layer schemes. In practice, longer spin‐up integrations help reduce drift, and the model state once stabilized can be assessed for suitability. Large drifts at the start of an integration have often been reduced by different tuning choices that either affect surface atmospheric fluxes or (more usually) ocean mixing (see section 2.2.1). Such retuning to reduce coupled model drift does not target the metrics that were used to hone the parameter settings of components very sensitive to model climate but not having a large direct impact on model climate, for example, modules for dust emission and lightning flash rate. Accordingly, the performance of those components will be worse in the simulations described in this paper than in atmosphere‐only simulations.

Note that the atmospheric component was tuned using the preindustrial f1 background ozone and aerosols. Upon switching to the f2 background, there was a slight drift in the coupled model. Prior to any historical runs with the f2 forcings, the coupled model was run a further 100 yr to reach a new quasi‐equilibrium.

We do not fine tune for an exact global mean surface temperature, since that is effectively precluded by the long spin‐up times and limited resources available. Similarly, no tuning was done for climate sensitivity or for performance in a simulation with transient forcing or hindcasts.

## 5 Climatology 1979–2014

As was seen in the results shown in Schmidt et al. (2014), the impact of interactivity in the aerosol or chemistry parts of the model have limited impacts on the climatologies. In addition, while in E2, there was a substantive difference in the composition fields between NINT and TCADI simulations, that is no longer the case in E2.1 (by design), though composition‐related interactivity may have an greater impact on the variability. We therefore only show the ensemble mean climatology from the standard NINT simulations (10 members for E2.1‐G, 5 members for E2.1‐H), in both spatial patterns, zonal and global means compared to updated observed climatologies for the satellite period (or as close as possible). All diagnostics are from the f2 historical simulations unless otherwise stated. We include the zonal mean diagnostics from the E2.1‐G f1 and f3 forcings ensembles for completeness where relevant, but the differences are mostly small. Note that the map projection uses Equal Earth (Šavrič et al., 2018) and that we now plot zonal means with an area weighted latitude axis to minimize visual distortion.

### 5.1 Global Mean Diagnostics

Table 2 summarizes a standard set of global mean diagnostics for the NINT versions of the GISS‐E2.1 models (with f2 forcings) and a comparison with up‐to‐date observations and previous model versions (Schmidt et al., 2014). Notable improvements are in the global mean temperature, precipitation, and sensible heat fluxes. The net radiative imbalance over this period is also in better comparison with updated estimates from the National Oceanographic Data Center (NODC). There are notable biases in total column water vapor (7% too high), and LW cloud forcing (some 20% to 25% too low, though still better than previously). Lower stratospheric water vapor is deficient, consistent with a too cold tropopause. The TOA radiative fluxes are tuned in preindustrial atmosphere‐only simulations and are therefore not truly predictive. Differences between the coupled models with different ocean modules are small compared to differences with the observations at the global mean level.

**Table 2:** Global Annual Ensemble Mean Model Features Over the Period 1979–2014 (1980–2004 for the E2 Models) and Key Diagnostics Compared to Observations or Best Estimates

Field	E2.1‐G	E2.1‐H	E2‐R	E2‐H	Observations
Surface air temp. (°C)	14.1	14.5	14.9	15.6	14.3 ± 0.5 (J)
Planetary Albedo	30.4	30.2	29.9	29.7	29.1 (C)/29.4 (SEA)
Cloud cover (%)	59.9	59.8	62	62	68 (SRK)
Precip. (mm day^−1^)	2.97	2.98	3.17	3.21	2.9 (G)
Snowfall (mm day^−1^)	0.24	0.23	0.19	0.17	0.18 (L08)/0.12 (SEA)
Atmos. water (mm)	26.7	26.8	23.8	24.0	24.9 (O)
Energy fluxes (W m^−2^):
TOA Absorbed Solar Rad.	236.9	237.5	239.5	240.3	240.2 (SEA)/239.4 (T)
TOA Outgoing Longwave Rad.	236.5	237.1	238.8	239.5	239.7 (SEA)/238.5 (T)
Surf. Abs. SW	161.5	161.9	169.5	170.1	165 (SEA)/169 (T)
Surf. Down. LW	345.8	347.4	341	344	345.6 (SEA)/343 (T)
Surf. Net LW (up)	50.5	50.7	56.9	56.9	52.4 (SEA)/57 (T)
Sensible heat flux	23.9	23.9	19.3	19.0	24 (SEA)/17 (T)
Latent heat flux	85.8	86.2	91.9	92.8	88 (SEA)/82 (T)
TOA SW cld. forcing	−48.8	−48.1	−48.9	−48.5	−45.4 (C)
TOA LW cld. forcing	21.1	21.1	18.8	19.0	25.9 (C)
TOA Net. Rad. Imb.	0.42	0.39	0.66	0.62	0.41 ± 0.03 (NN)
Trop. lower strat. water vapor minima (ppmv)
	3.0	2.8	4.5	4.4	3.8 ± 0.3 (D)
Zonal mean tropopause temp. (min., DJF) (°C)
	−81	−82	−80	−80	−80
Hadley Circ. (10^9^ kg s^−1^) (DJF)
	205	207	206	208	170–238 (S)

*Note*. Cloud cover is estimated based on clouds with optical thickness >0.1. J: Jones et al. (1999) with updates; C: CERES EBAF Ed4.1 (Loeb et al., 2019); T: Trenberth et al. (2009) and updates; G: GPCP V2.3/TRMM TMPA V7 (Huffman et al., 2007, 2009); O: Obs4MIPs; NN: derived from NOAA NODC ocean heat content data; D: Dessler (1998); L08: Liu (2008); S: Stachnik and Schumacher (2011); SEA: Stephens et al. (2012); SRK: Stubenrauch et al. (2013).

### 5.2 Radiation and Clouds

**Figure 3:**
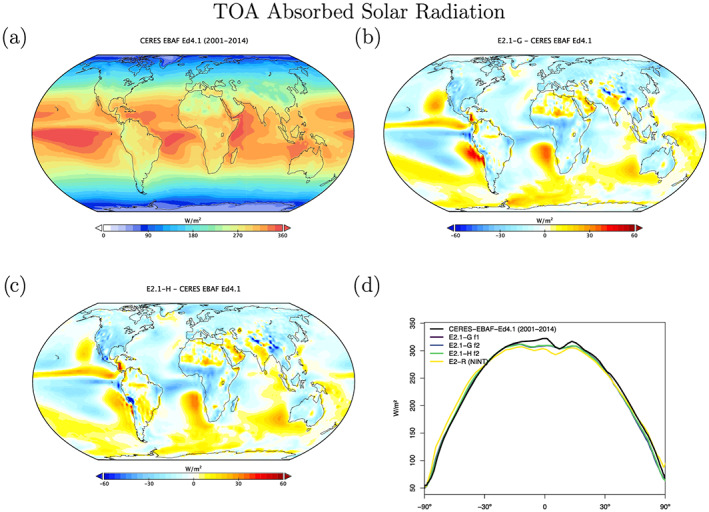
(a) Annual climatology of TOA Absorbed Solar Radiation (W m^−^2) in CERES EBAF Ed4.1 (Loeb et al., 2019). (b and c) Difference of E2.1‐G and E2.1‐H from the observations. (d) Absolute Zonal means, including E2.1‐G (f1 and f2), E2.1‐H, and the earlier model version, E2‐R.

**Figure 4:**
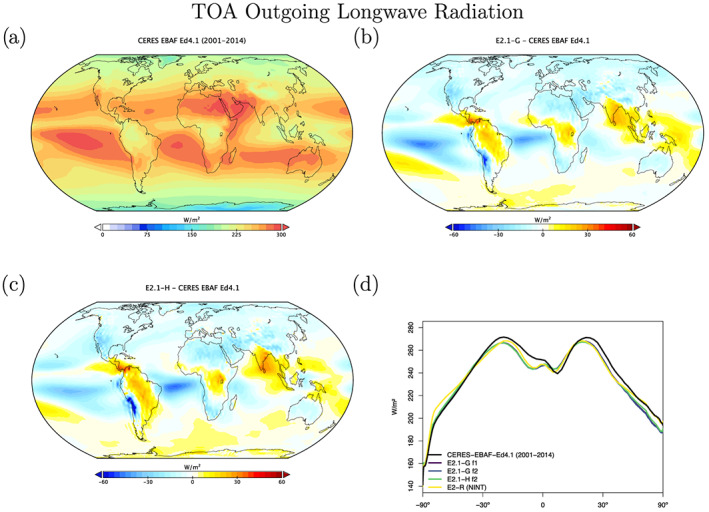
Annual climatology of TOA Outgoing Longwave Radiation in data and models, as in Figure 3.

**Figure 5:**
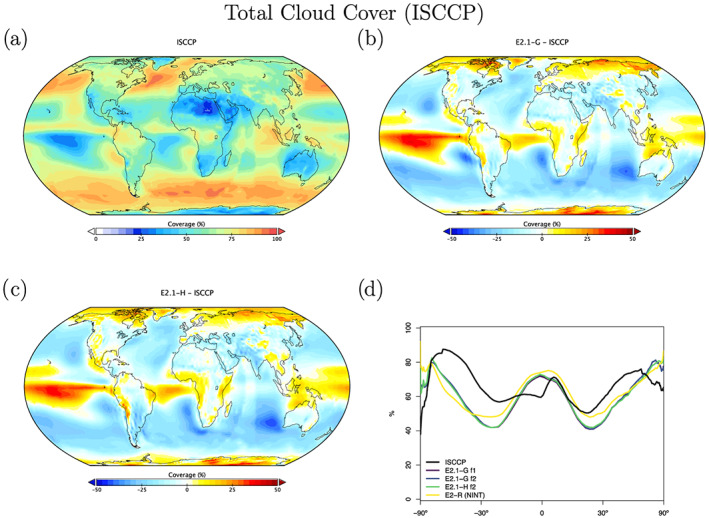
Annual climatology of total cloud cover as seen by ISCCP‐H, figure description as in Figure 3.

**Figure 6:**
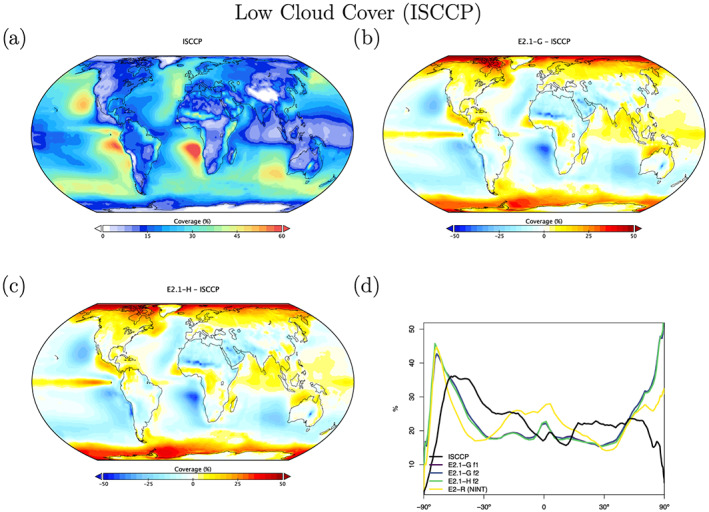
Annual climatology of low cloud cover as seen by ISCCP‐H, figure description as in Figure 3.

**Figure 7:**
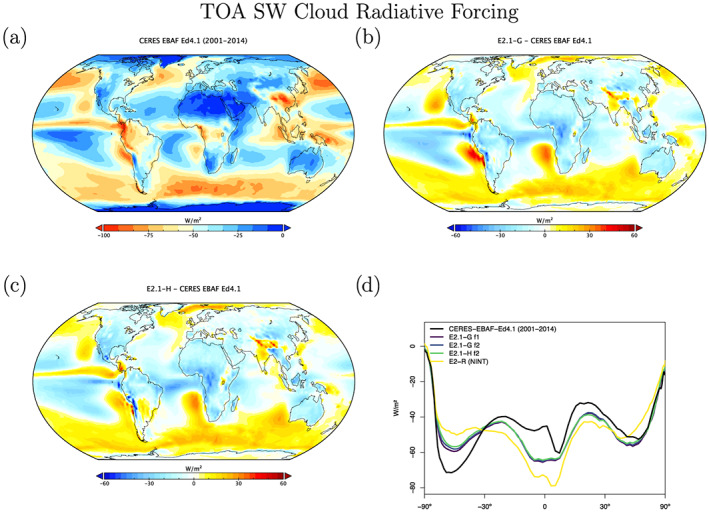
Annual climatology of short wave cloud radiative forcing, figure description as in Figure 3.

Radiation diagnostics are compared to the latest balanced CERES product (EBAF Ed4.1) (Loeb et al., 2019). Improvements since E2 are clearest in the Southern Ocean, where excessive SW absorption has been greatly ameliorated, and also in the tropics, although obvious biases associated with the marine stratus regions in the eastern ocean basins still exist (Figures 3 and 4). Notably, the sign of the biases in the Arctic have changed in SW absorption. There is a lack of cross‐equatorial asymmetry (which is clear in the observations), with the southern tropics characterized by excessive water vapor and cloud forcing, evidence of a remnant double‐ITCZ (Intertropical Convergence Zone) bias. In the Southern Ocean latitudes, both total and low cloud cover are increased in E2.1 compared to E2, reducing the bias (Figures 5 and 6). Note that Southern Ocean estimates of TOA absorbed solar radiation (Figure 3) are somewhat better constrained than SW cloud radiative forcing (Figure 7).

**Figure 8:**
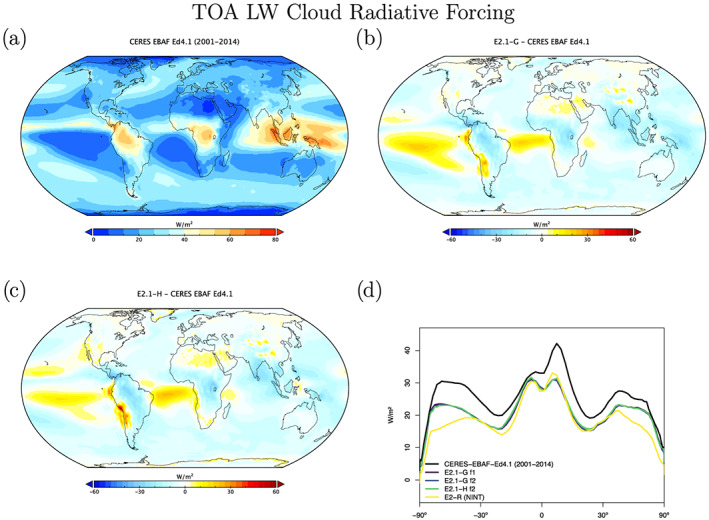
Annual climatology of long wave cloud radiative forcing, figure description as in Figure 3.

**Figure 9:**
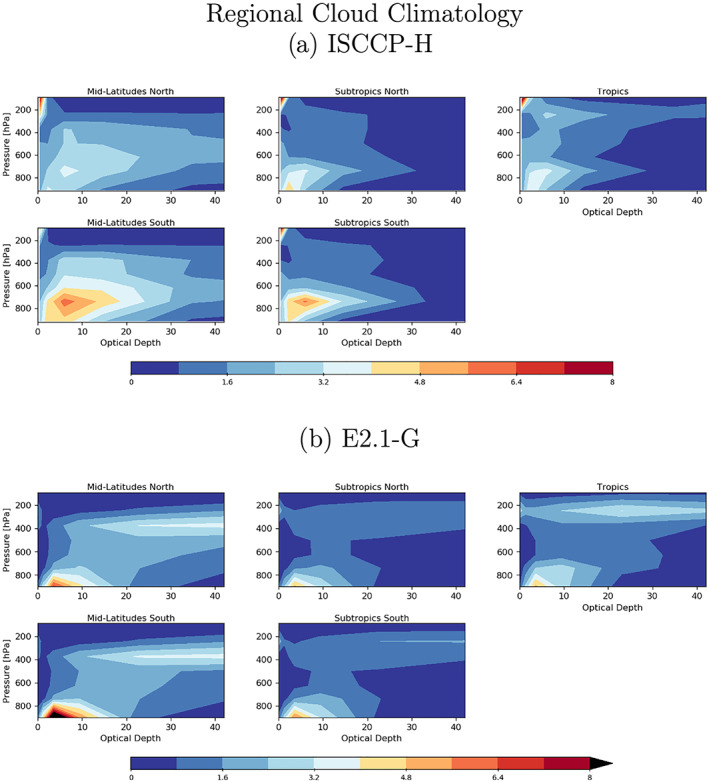
Climatology of cloud occurrence as a function of optical depth and pressure for five latitudinal bands as seen by ISCCP (60–30°N, 30–15°N, 15°N to 15°S, 15–30°S, and 30–60°S). (a) Data from ISCCP‐H (Young et al., 2018). (b) Data from the ensemble mean E2.1‐G results. (Results from E2.1‐H are indistinguishable).

Cloud fraction observations have been upgraded to the ISCCP‐H product over 1984–2014 (Young et al., 2018). The overall patterns in E2.1 are slightly improved in the tropics and midlatitudes, but the persistent biases (in the marine stratus regions) remain clear (Figures 5 and 6). The bias in low cloud over sea ice regions may however be an artifact. The improvements are clearer in the SW CRF diagnostic (Figure 7), and in the high latitudes at least for the LW cloud radiative forcing, which remains overall too low (except in the erroneously cloudy tropical mid‐Pacific (Figure 8). The cloud top pressure/cloud optical depth histograms (Figure 9) show that the model has improved its “too few‐too bright” low cloud problem, as low cloud cover has increased and optical thickness has decreased in relation to the E2 version (Schmidt et al., 2014).

Comparisons of an earlier E2.1 version with active‐sensor satellite observations (not shown) confirms an improvement of the low cloud cover in the high latitudes and over the trade wind regions while large biases remain over the stratocumulus regions in the tropics and subtropics. This low cloud bias might alter the strength of the low cloud feedbacks in response to global warming (Cesana et al., 2019; Marvel et al., 2018; Zhou et al., 2016). The large high‐cloud positive bias found in E2 (Cesana & Waliser, 2016) has been mostly removed except in the Southern Hemisphere tropics, where the overestimate of total cloud cover (Figure 5) comes from an excess of very high clouds (above 16 km), which are not present in satellite observations. The amount of E2 supercooled water cloud relative to ice cloud was underestimated on average (Cesana et al., 2015), while E2.1 has the opposite bias (Figure 1). In a warming world, a shift from ice crystals to liquid water droplets results in brighter clouds; which gives rise to a (negative) cloud‐phase feedback (Ceppi et al., 2016; Tan et al., 2016). Models that start with excessive cloud ice have the potential to exaggerate this feedback; thus, the cloud‐phase feedback might be underestimated in E2.1 while it was likely overestimated in E2, partially contributing to the higher climate sensitivity (see section 6).

**Figure 10:**
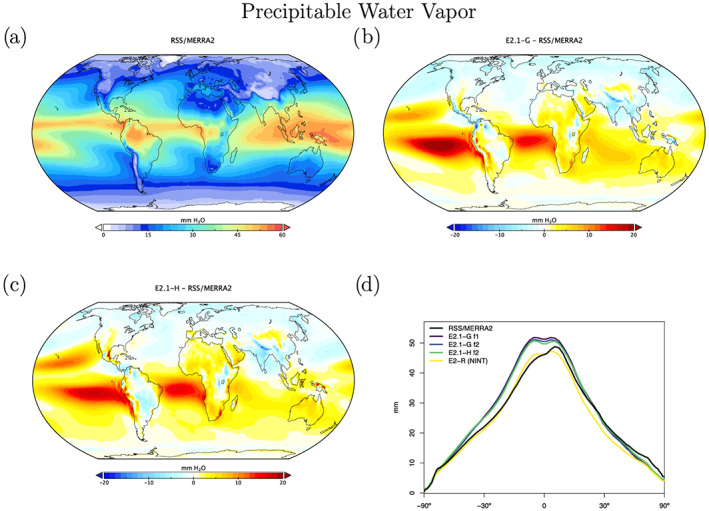
Annual climatology of precipitable water vapor, figure description as in Figure 3. Data derived from a blend of RSS and MERRA2 products over ocean and land respectively.

**Figure 11:**
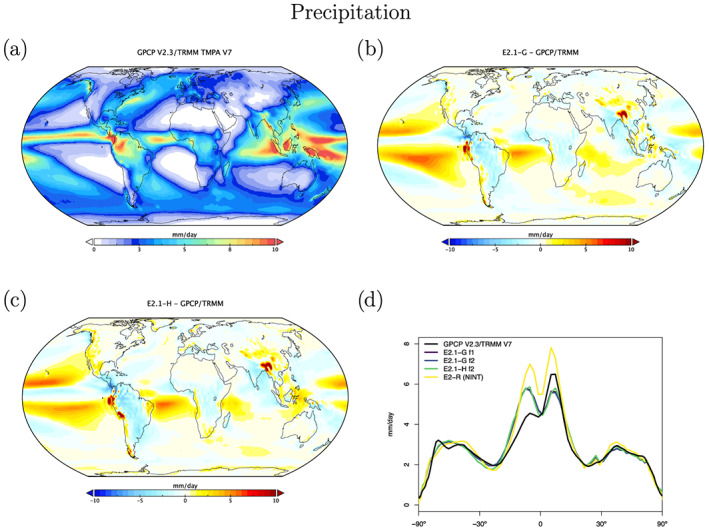
Annual climatology of precipitation. Figure description is as Figure 3.

Atmospheric hydrological observations come from two blended data products via the Obs4MIPS archive (Ferraro et al., 2015; Gleckler et al., 2011; Teixeira et al., 2014). The precipitable water vapor is a blend of the Remote Sensing Systems (RSS) product over ocean (Wentz & Schabel, 2000; Wentz et al., 2007) and MERRA‐2 (over land) from the CREATE‐MRE project (Potter et al., 2018) while the precipitation product is a blend of TRMM satellite estimates over ocean (Adler et al., 2009; Huffman et al., 2007) and GPCP (Huffman et al., 2009) Version 2.3 satellite‐gauge calibrated precipitation over land. Precipitable water vapor discrepancies (Figure 10) are larger than in E2 in the tropics, where the lack of asymmetry is readily apparent. The largest biases in water vapor coincide with the excessive LW CRF. This is also consistent with overall precipitation biases (Figure 11), which show a classic double‐ITCZ problem in the Pacific, although one that is diminished in magnitude compared to E2. Excessive land precipitation in the Western Pacific Warm Pool has also been greatly ameliorated. Note too, that part of the reduced bias in rainfall is due to upgrades in the observational product.

**Figure 12:**
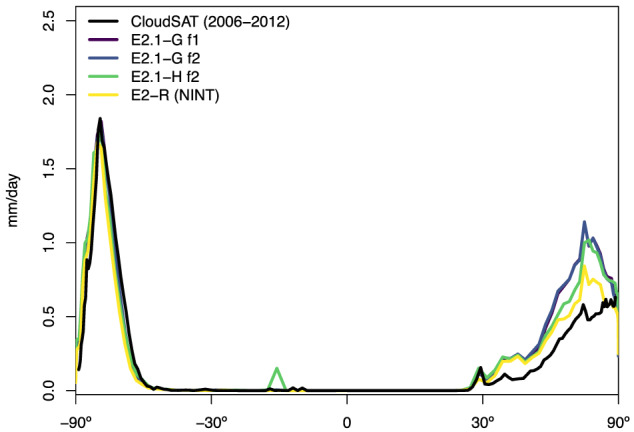
Annual climatology of snowfall compared to CloudSAT data (Liu, 2008).

Snowfall biases are noticeable in the zonal mean (Figure 12), particularly in the Arctic, where excessive snowfall is related to wintertime cold biases in both models.

### 5.3 Satellite‐Derived Atmospheric Temperatures

**Figure 13:**
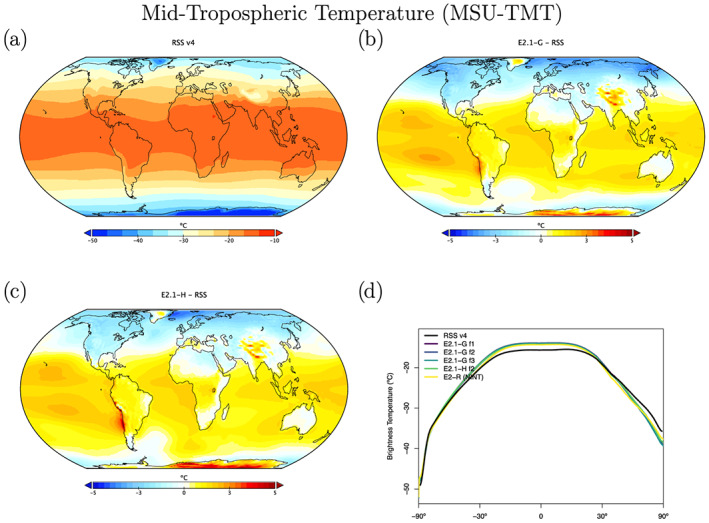
Annual climatology of MSU TMT. Observational data comes from RSS (1979–2014) (Version 4.0) (Mears & Wentz, 2016). Figure description is as Figure 3 with the addition of the zonal mean results for the E2.1‐G (f3) configuration.

**Figure 14:**
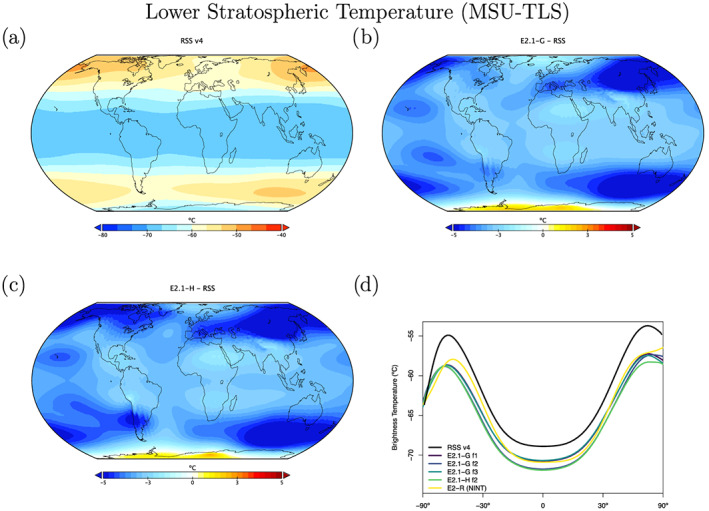
Annual climatology of MSU TLS. Observational data comes from RSS (1979–2014) (Version 4.0) (Mears & Wentz, 2016). Figure description is as Figure 13.

**Figure 15:**
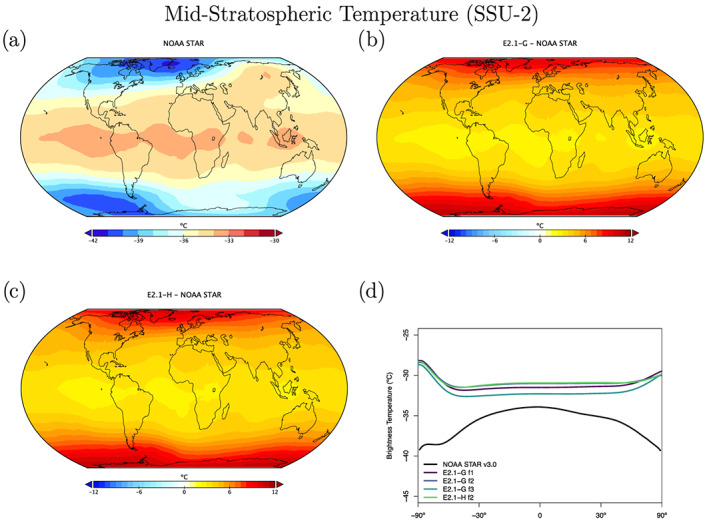
Annual climatology of SSU Channel 2. Observational data comes from NOAA STAR (1979–2014) (Version 3.0) (Zou & Qian, 2016). Figure description is as Figure 13, with the omission of the E2 diagnostics which were not calculated at the time.

The structure of temperature through the atmosphere plays a large role in defining fingerprints of climate change forcings, and so we compare the models to the Microwave Sounding Unit (MSU) and Stratospheric Sounding Unit (SSU) 1979–2014 brightness temperature climatologies (Figures 13, 14, 15). We highlight results from the midtroposphere (TMT), the lower stratosphere (TLS), and middle stratosphere (SSU Channel 2), which have global weightings centered on 600, 70, and 4 hPa, respectively (though with substantial tails) (Mears & Wentz, 2016; Zou & Qian, 2016). We use a static weighting function to estimate the channels, which though slightly less accurate than a radiative transfer calculation that takes into account surface emissivity, atmospheric water vapor, and temperature profiles (Shah & Rind, 1995), does not produce significantly different results.

Starting with MSU‐TMT (Figure 13), the models show a notable warm bias in the tropics and subtropics, indicating a slightly less steep lapse rate in the troposphere than observed, and a cold bias in the northern high latitudes. Warm biases over high topography may be an artifact of the diagnostic comparison.

In the lower stratosphere (Figure 14), the models are anomalously cold, though partially the poorer comparison to observations since E2 is related to an warmer climatology in the latest RSS Version 4.0 (Mears & Wentz, 2016). The middle and upper stratosphere (as illustrated by the SSU‐2 channel; Figure 15) is too warm—particularly in the polar regions. This overall pattern of stratospheric temperature error is consistent with, but not completely explained by, a too strong Brewer‐Dobson circulation in this relatively low‐top model.

### 5.4 Surface Fields

**Figure 16:**
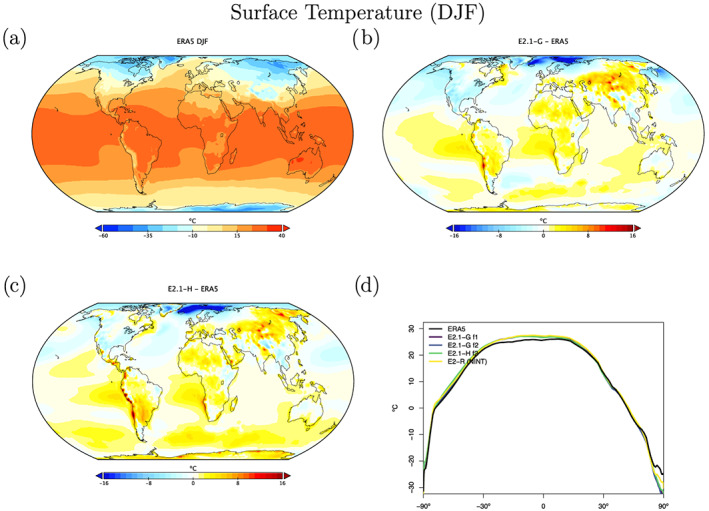
DJF climatology of surface air temperature. Figure description is as Figure 3.

**Figure 17:**
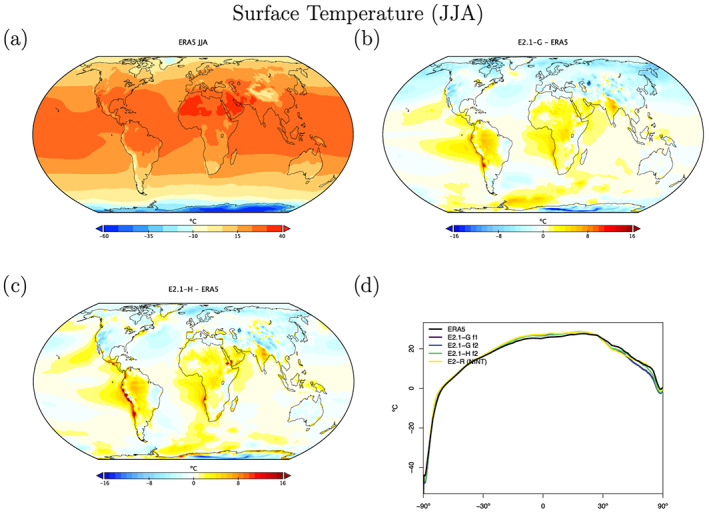
JJA climatology of surface air temperature. Figure description is as Figure 3.

Surface field climatological observations are taken from the European Centre for Medium Range Weather Forecasting Re‐Analysis 5 (ERA5) (Copernicus Climate Change Service (C3S), 2017), which is a well‐validated and spatially complete data set (Hersbach et al., 2020). Overall biases in E2.1 for the surface temperature fields (Figures 16 and 17) are similar to CMIP5, though the magnitude of errors in the Southern Ocean are notably reduced (consistent with the improvements of cloud and radiation diagnostics discussed above). Land errors are reduced, though the winter cool bias in the Arctic is slightly increased.

**Figure 18:**
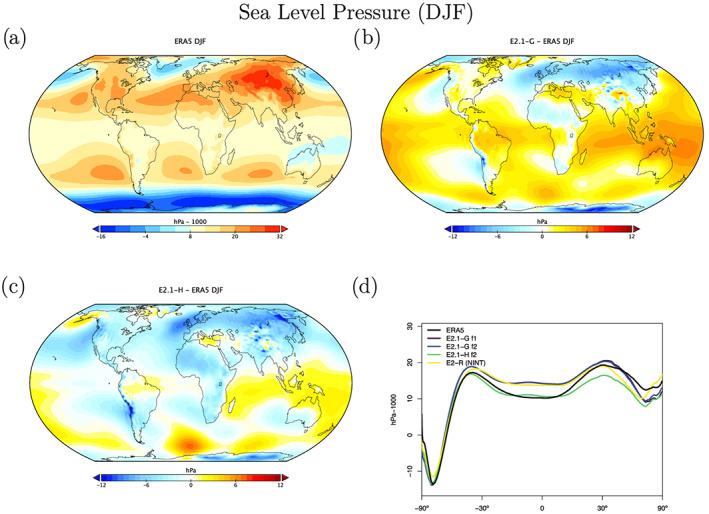
DJF climatology of sea level pressure (including water vapor mass in the diagnostic, even though it is not seen by the dynamics). Figure description is as Figure 3.

**Figure 19:**
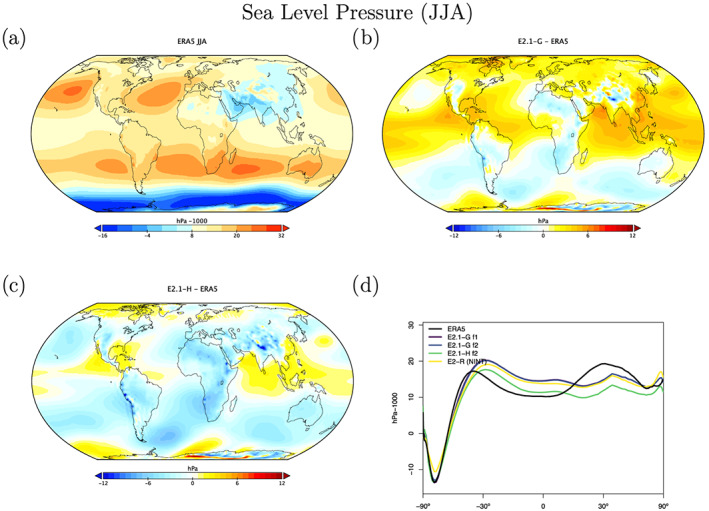
JJA climatology of sea level pressure. Figure description is as Figure 18.

**Figure 20:**
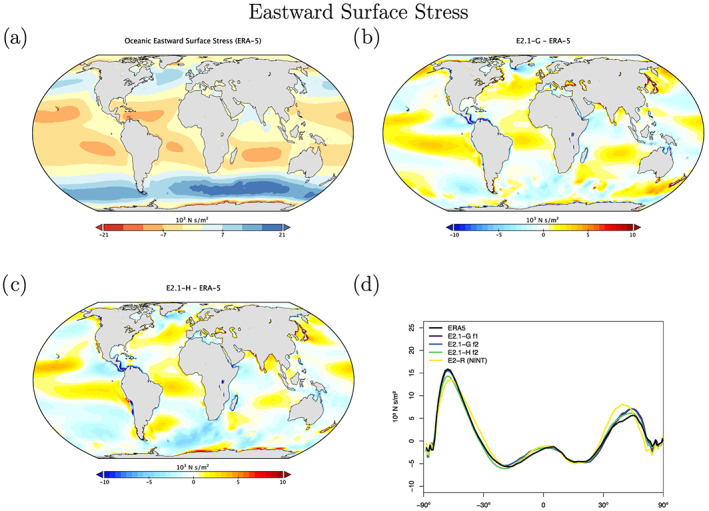
Annual climatology of oceanic Eastward surface stress. Figure description is as Figure 3.

Sea level pressure biases are quite different between the two ocean model versions (Figures 18 and 19), with E2.1‐G having a larger positive bias in the tropics than in E2.1‐H. This is partially explained by the higher than observed water vapor in the models, and the topographic change made in the HYCOM land‐ocean grid which increased surface pressure over land (with a corresponding ocean decrease through conservation of atmospheric mass). In the northern summer, both models fail to generate as large an extra‐tropical gradient as observed. However, the overall pattern of surface wind stress is improved from E2 (Figure 20), with notably more poleward maxima in the middle‐to‐high latitudes. There remains a westward bias in the eastern tropical Pacific.

The wind stress improvements arise from a combination of atmospheric process affecting the SLP patterns and coupled processes that affect the surface latitudinal temperature gradients. The improvements in ocean heat transports (Figure 23) in both hemispheres (but particularly in the Southern Ocean) push the storm tracks poleward and increase the midtroposphere temperature gradient, sharpening the maxima. Even in atmosphere‐only simulations this is improved though, indicating that the boundary layer and cloud improvements on their own are positively impacting the SLP and wind stress.

Runoff from the major rivers can be compared to observational data (Fekete et al., 2001) (Table 3). In the tropics, runoff is severely deficient in the Amazon basin and African rain forests (due to insufficient rainfall) and skill has not increased compared to earlier model versions. High latitude rivers are, however, more consistently modeled. Skill in reproducing the seasonal cycle of river discharge varies with latitude. Discharge from the tropical rivers is too low throughout most of the year, with large discrepancies in Southern Hemisphere summer and fall. The amplitude and phase of discharge from midlatitude rivers is consistent with observations. The peak of modeled high latitude river discharge tends to be too low, and too broad, and occurs later in the season than in observations.

**Table 3:** Annual Mean Discharge From Selected Rivers (km^3^ month^−1^)

River	E2.1‐G	E2.1‐H	E2‐R	E2‐H	Observations
Amazon	241–262	280	198–236	229–300	545
Congo	20–23	36	35–69	41–82	106
Brahmaputra‐Ganges	118–135	81	68–86	110–140	105
Yangtze	104–111	111	85–100	191‐210	78
Lena	44–46	41	32–34	29–31	40
Ob	50–53	38	47–52	80–89	33
St. Lawrence	54–58	35	53–55	27–28	29
Mackenzie	23–24	29	28–29	31	24

*Note*. Ranges given across the climatological means over 1979–2014 for the E2.1‐G ensemble (1979–2005 for E2‐R/H), and ensemble mean for E2.1‐H. Observations from Fekete et al. (2001).

### 5.5 Ocean

We focus here on the diagnostics that most impact the coupled simulation and are straightforwardly comparable to observations. More detailed description and analysis of E2.1 ocean circulation and structure will be presented elsewhere.

**Figure 21:**
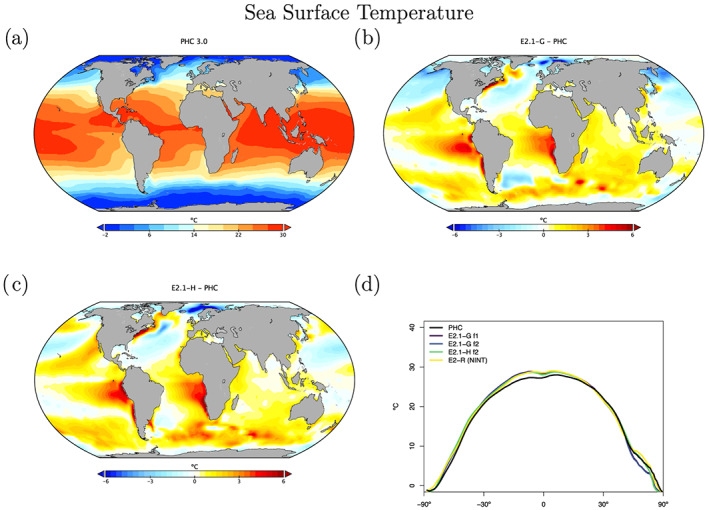
Annual climatology of sea surface temperature compared to the PHC 3.0 product (updated from Steele et al., 2001). Figure description is as Figure 3.

SST biases (Figure 21) are still dominated by the errors in the marine stratus regions and Arctic biases are colder than before. Overall, tropical temperatures are slightly warm, particularly in the southern tropics, which is consistent with the errors in precipitable water vapor, clouds and radiation seen above. Remarkably, the two ocean models exhibit generally similar patterns of bias.

**Figure 22:**
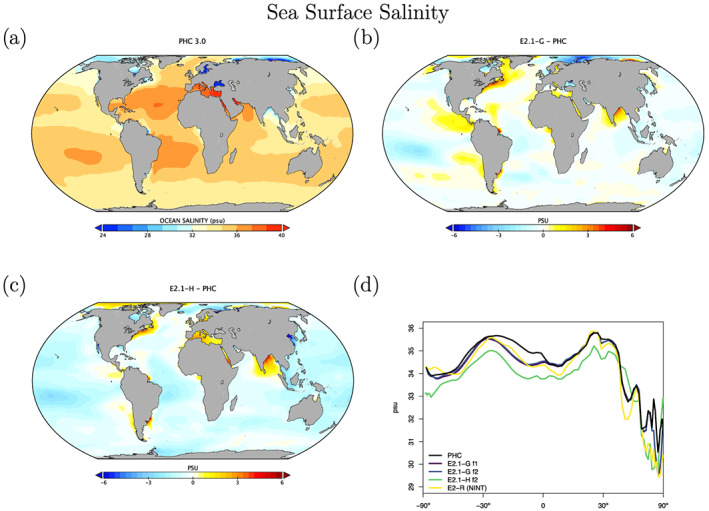
Annual climatology of sea surface salinity (PSU) compared to the PHC 3.0 product. Figure description is as Figure 3.

Salinity biases in E2.1‐G are far smaller than in E2‐R, particularly in marginal seas, but also in the open ocean (Figure 22). Clear positive biases are obvious near major river mouths (consistent with insufficient river outflow seen in Table 3).

For HYCOM, the biases in surface salinity (Figure 22c) have been totally reversed, in part due to the correction to virtual salt fluxes, from a large excess salinity in E2‐H, to an overall underestimated salinity in E2.1‐H, though with a reduced overall error. Arctic biases are noticeably reduced, possibly associated with the implementation of the BP ice thermodynamics.

**Figure 23:**
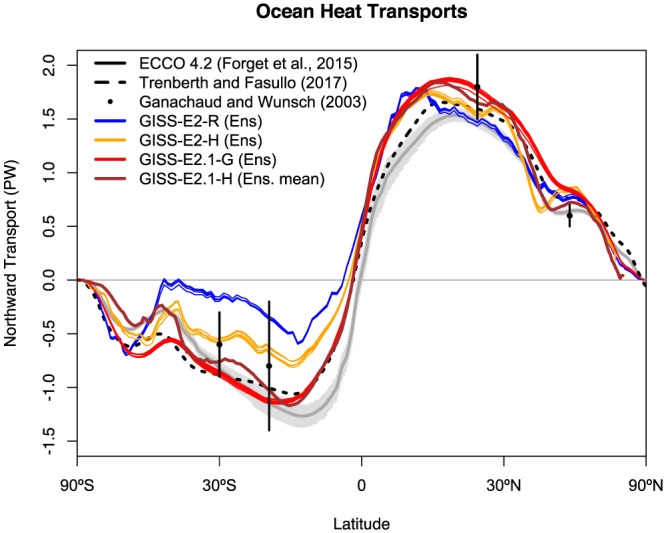
Annual mean global northward ocean heat transports. Comparisons of the models with mean estimates from 1992–2011 from the ECCO ocean state estimate (v4 release 2) with 95% confidence intervals on the mean derived from the interannual variability (Forget et al., 2015; Forget & Ferreira, 2019), imputations from reanalyses (Trenberth & Fasullo, 2017) (2000–2016), and oceanographic estimates (Ganachaud & Wunsch, 2003).

Ocean transports are also greatly improved, notably the Drakes Passage where offsets to the observed transport are much less than previously in both models (Table 4). Fluxes through the Gulf Stream and Kuroshio Current are reasonable, but slightly higher than inferred from observations. The mass and heat transports at 26°N from the north. Atlantic overturning circulation in E2.1‐H are in good agreement with direct observations (Johns et al., 2011; McCarthy et al., 2015; Smeed et al., 2019), but larger in E2.1‐G. Poleward heat transports peak above 1 PW at ∼20°N; this is significantly higher than the estimates derived from a ocean state estimation approach (Forget & Ferreira, 2019) (Figure 23), but in reasonable agreement with direct heat flux estimates (Ganachaud & Wunsch, 2003). Poleward transports in the southern oceans in E2.1‐G are much more consistent with both direct measurements and ocean state estimates.

**Table 4:** Selected Ocean Mass (Sv) and Heat (PW) Fluxes

Diagnostic	E2.1‐G	E2.1‐H	E2‐R	E2‐H	Observations
N. Atl. MOC (Max)	27.2	20.4 ± 0.3	27.2 ± 0.7	24.5 ± 0.8	—
N. Atl. MOC (26°N)	24.8 ± 0.4	17.8 ± 0.3	18.4 ± 0.3	22.4 ± 0.6	≈18 (R19)
Atl. Heat (26°N)	1.21 ± 0.01	1.06 ± 0.01	0.97 ± 0.01	0.99 ± 0.02	1.3 ± 0.4 (J11)/0.88 ± 0.01 (F19)
ACC (Drake Pass.)	150 ±1	178 ±1	254 ± 1	192 ± 2	130 (P88)/173 (D16)
Gulf Stream	55 ± 1	48.2 ± 0.3	49 ± 1	39.8 ± 0.8	≈35 (R11)
Kuroshio	49 ± 1	67 ± 2	64 ± 1	71.7 ± 0.5	≈57 (I01)
Bering Str.	0.16 ± 0.003	−0.55 ± 0.01	0.16 ± 0.01	0.45 ± 0.01	0.8 ± 0.2 (W05)
Indonesian throughflow	18.9 ± 0.2	18.4 ± 0.2	11.5 ± 0.2	17.6 ± 0.3	15 (S09)

*Note*. Range is standard deviation of the 1979–2014 average from five ensemble members for each configuration. Observations: R19: (McCarthy et al., 2015; Smeed et al., 2019) (estimate at 26°N); P88: Petersen (1988); D16: Donohue et al. (2016); J11: Johns et al. (2011); R11: Rayner et al. (2011); I01: Imawaki et al. (2001); W05: Woodgate et al. (2005); S09: Sprintall et al. (2009); F19: Forget and Ferreira (2019).

Sensitivity experiments with a reduced tidal mixing efficiency in E2.1‐G suggested that tuning of this parameter could match the target Atlantic overturning transport metric at 26°N and the Forget and Ferreira (2019) heat transport there, but with the penalty of unacceptably increasing cold biases in northern midlatitudes and the Arctic. Such compromises will be revisited in future model versions having improved cloud radiative forcing and atmospheric transports. Ocean‐only experiments with an E2.1‐G prototype (Romanou et al., 2017) indicate that its CFC uptake is best matched in configurations having weaker AMOC magnitudes than those realized here, which has implications for heat and carbon uptake.

### 5.6 Cryosphere

**Figure 24:**
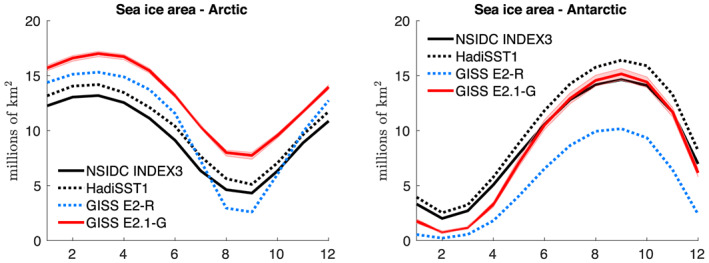
Annual climatology of sea ice area in both hemispheres in E2‐R (blue dashed) and E2.1‐G (red). Observational data comes from NSIDC (1979–2014), after correction for the Arctic polar “hole” (Fetterer et al., 2011) and HadISST1 (1979–2014) (Rayner et al., 2011). The ensemble mean climatology is plotted for E2‐R (1979–2012) and E2.1‐G (1979–2014, with spread across E2.1‐G ensemble members in pink).

**Figure 25:**
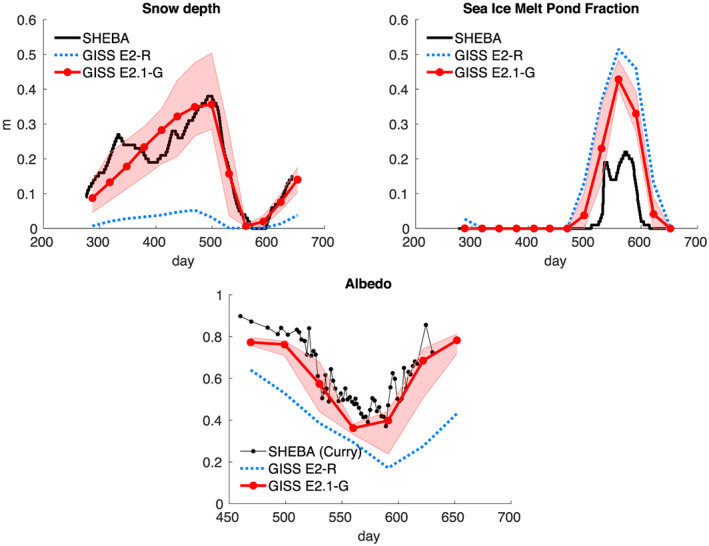
Spot comparisons of the E2‐R (blue dashed) and E2.1‐G (red) simulations against the SHEBA measurements for snow depth, melt pond fraction and albedo (Curry et al., 2001). Ensemble spread for E2.1‐G is in pink.

Figure 24 shows that the amplitudes of the seasonal cycle of sea ice extent have improved in both hemispheres in E2.1‐G. For the Arctic, changes (1) and (3) described in section 2.3 reduce summer melt and winter growth, and the resulting increase in snow depth and albedo compares favorably to SHEBA data (Figure 25). In the Antarctic, improvements are largely due to a more stratified ocean and an associated reduction of upward mixing of warm subsurface water, as opposed to changes in sea ice physics or properties (as has been the case previously; Liu et al., 2003). Sea ice distributions in E2.1‐H are broadly similar, though warmer conditions in the North Pacific (Figure 17) are associated with less anomalous sea ice there.

**Figure 26:**
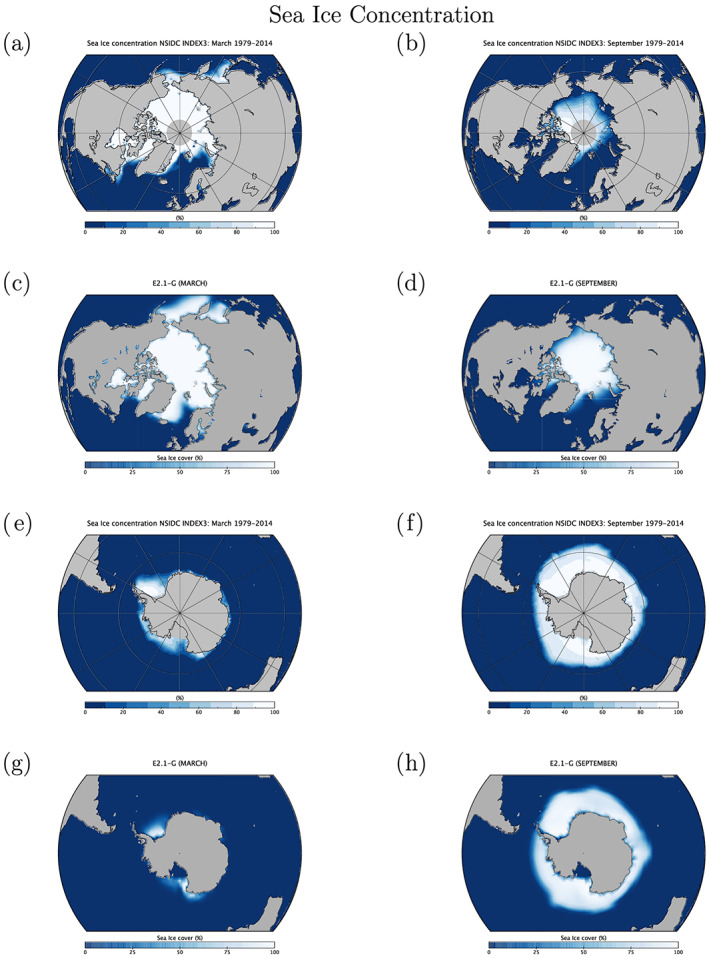
Sea ice concentration (%) for March (left column) and September (right column) in the NSIDC observations and E2.1‐G simulations. Panels (a)–(d) are for the Arctic, and (e)–(h) Antarctic. E2.1‐H results are similar.

Brighter middle and high latitude clouds in E2.1 (Figure 3) cool surface temperatures and aid ice formation, driving deficient Antarctic ice closer to observed but increasing the Arctic excess. Figure 26 presents the spatial structure of the concentration biases. In the Antarctic, the winter ice edge reaches approximately the correct latitude, but summertime conditions only permit ice in limited areas. Derivatives with respect to latitude in Figure 23 indicate that the modeled ocean currents lose too much heat to the atmosphere at latitudes surrounding the Arctic, leaving insufficient warmth to prevent wintertime ice formation in the North Pacific and Barents Sea sectors. In addition to too bright clouds (Figure 3), this excess heat loss also has a contribution from a free‐tropospheric cool bias over the Northern extratropics (Figure 13), which also exists in atmosphere‐only simulations to a lesser extent (not shown), and coarse ocean resolution, which reduces the speed of warm (boundary) currents, particularly those entering the GIN and Barents seas known to be important for regional heat budgets (Smedsrud et al., 2010).

### 5.7 Model Internal Variability

As model processes have become more sophisticated and the base climatology has become more realistic, the representation of the patterns of internal variability has also improved. We focus here on ENSO, the Pacific Decadal Oscillation (PDO), and the MJO because the improvements over previous models have been most dramatic. Notably, while the MJO was a specific target for improvement through the model development process, the changes in ENSO and PDO patterns emerged as part of the overall improvement in skill.

**Figure 27:**
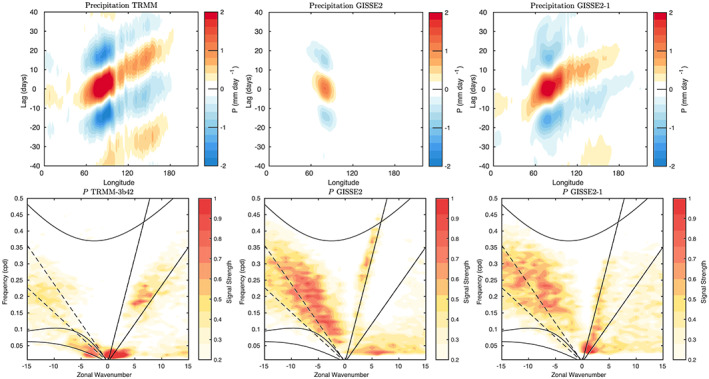
Comparison of MJO signals and propagation in the TRMM data (release 3b24), (Iguchi et al., 2000) and in E2‐R and in E2.1‐G simulations. (top row) Hovmöller plots of MJO propagation. (bottom row) Wheeler‐Kiladis diagrams for tropical wave motion (Wheeler & Kiladis, 1999). Figures courtesy of Ángel Adames.

The MJO improvement is highlighted in Figure 27, where the lack of MJO‐related activity and propagating features in the Pacific in E2‐R was very clear in comparison with an analysis of the TRMM data. However, in E2.1‐G, the improvement in propagation and in the wavenumber/frequency plot (Wheeler & Kiladis, 1999) is evident.

**Figure 28:**
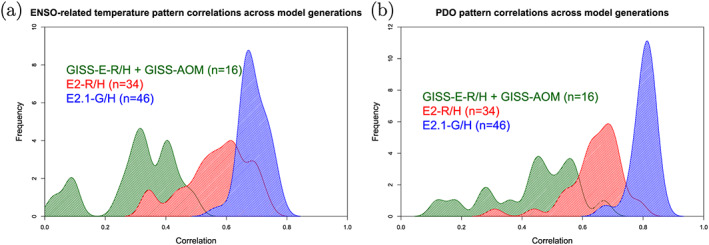
Improvement of modeled spatial correlations of the temperature patterns associated with (a) ENSO and (b) the PDO, to the observed patterns for each GISS model generation (CMIP3 (green) to CMIP5 (red) to CMIP6 (blue)). Calculations via the Climate Variability Data Portal (CVDP) (Phillips et al., 2014), using surface air temperature correlations to the Nino3.4 index and the leading PC of the detrended North Pacific SST decadal variability (Mantua et al., 1997) derived from Berkeley Earth Global Mean Surface Temperature (Rohde et al., 2013) and ERSSTv5 SST (Huang et al., 2017) over the period 1900–2005.

**Figure 29:**
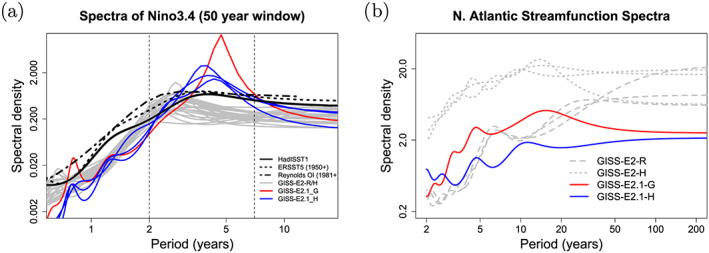
(a) Spectra of Nino3.4 variability in 50 yr segments from the PI controls compared to various observational products. Improvement of pattern correlations of the PDO to the observations over GISS model generation (from CMIP3 to CMIP6). (b) Spectra of variability in the North Atlantic annual mean maximum stream function (derived from a detrended 1,000 yr of PI control simulation).

For the longer term tropical modes, ENSO and the PDO, there have been large improvements in the patterns of associated temperature variability (Figure 28) across CMIP generations, and particularly since CMIP5. However, that improvement must be tempered by a recognition that the spectral signature of ENSO has not improved (Figure 29a). In all versions of E2, there was insufficient overall variance, and in particularly a deficit in variability at 3–7 yr (overall standard deviations in the Nino3.4 index were 0.60°C for E2‐R and 0.67°C for E2‐H, compared to ∼1°C in the ERSST5 observations; Huang et al., 2017). However, in E2.1‐G and E2.1‐H the 2 to 4 yr variability is now too strong. The overall Nino3.4 standard deviation is too strong (1.2°C) in E2.1‐G though still too low in E2.1‐H (0.75°C). The excessive variance in E2.1‐G impacts the interannual variability worldwide, even for the global mean, leading us to increase the number of ensemble members to 10 in the historical simulations in order to be better able to assess the forced responses.

The larger overall ENSO variability and unrealistically peaked spectral signature in E2.1‐G relative to E2.1‐H suggest that ocean interior structure and damping mechanisms exert as much influence as atmospheric processes. Some of the latter have been quantified in feedback form for E2.1‐G following Figure 7 in Bellenger et al. (2014). Specifically, the wind stress (positive) feedback is 9.8 ×10^−3^ N m^−2^ °C^−1^, 20% weaker than in ERA40, and the surface‐flux (negative) feedback is −12.5 W m^−2^ °C^−1^, 30% weaker than observed. In a sensitivity test (similar to one reported in Rind et al., 2020), we applied a change to the atmospheric convection scheme that led to reduced ENSO amplitude and a shift of the peak to shorter periods. Both of the feedback coefficients are significantly smaller in that simulation, suggesting that its ENSO improvement occurred for the wrong reasons, and overall model skill was not enhanced. This remains an active area of model testing, although we anticipate that it will require a substantial improvement of marine stratus biases (as a function of increased vertical resolution and better moist physics) before specific tuning for the correct ENSO feedbacks will become worthwhile.

In the North Atlantic, where decadal and longer period variability is associated with the overturning stream function, there are mixed changes. There is greater variability at 8–15 yr for E2.1‐G compared to E2‐R, but significantly less variability in E2.1‐H compared to E2‐H (Figure 29b). The standard deviation of the detrended annual stream function maximum at 26°N is 1.7 Sv for E2.1‐G, and 0.8 Sv for E2.1‐H. This can be compared to the interannual variability in the observed meridional overturning circulation at the same latitude of ∼1.3 Sv (McCarthy et al., 2015; Smeed et al., 2019).

### 5.8 Summary Statistics

**Figure 30:**
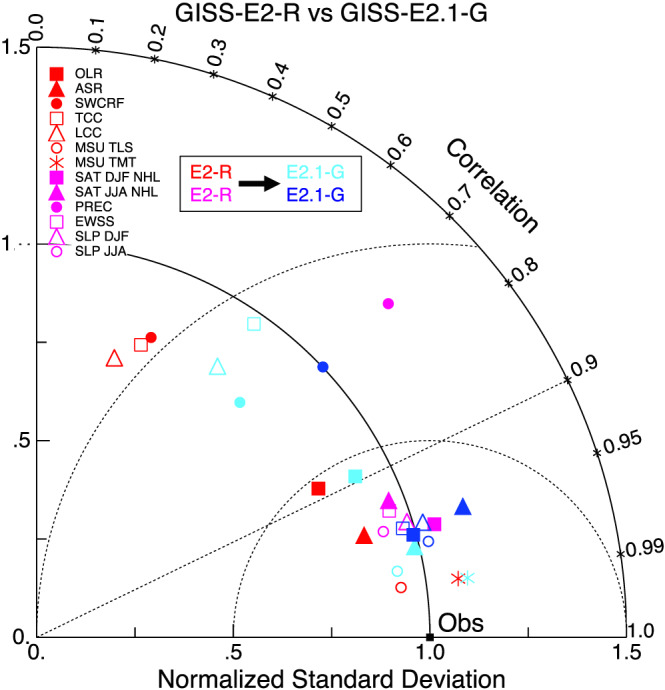
Summary Taylor diagram for selected quantities showing the difference in performance for E2.1‐G (light and dark blue symbols) compared to E2‐R (red and purple) for different fields. The change in each field can be tracked by going from the red (purple) symbol to the corresponding light blue (dark blue) one. Data sources: CERES EBAF 4d1b: Outgoing Longwave Radiation (OLR) and Absorbed Solar Radiation (ASR) (60°S to 60°N); RSS v4 MSU‐TMT and MSU‐TLS; ISCCP‐H Total Cloud Cover (TCC), Low Cloud Cover (LCC) (60°S to 60°N), ERA‐5 oceanic Sea Level Pressure (SLP) (DJF and JJA), SAT over Northern Hemisphere Land (NHL) (DJF and JJA), and oceanic Eastward Surface Stress (EWSS); TRMM/GPCP Precipitation.

We are interested both in how model evolution affects skill scores and also in how the GISS model compares to similarly functional models in the CMIP5 and CMIP6 ensembles. Improvements across the board are seen in the standard large‐scale climatological metrics presented in the Taylor diagram comparing E2‐R with E2.1‐G f2 (Figure 30) (differences with other configurations are slight). The improvements are largest in fields that were the worst performing in CMIP5 (clouds and precipitation), though still positive for even well‐simulated fields. As in previous papers, we can calculate an Arcsin‐Mielke score (between 0 and 1) (Watterson, 1996) for a suite of standard variables (Table 5). These reflect the same general tendencies. Differences between the f1 and f2 ensembles are barely perceptible (except for MSU‐TLS, which is a little better in the f1 ensemble).

**Table 5:** Arcsin‐Mielke Scores Across Model Configurations for Selected Fields as Referenced Above (see Figure 30 for the Field Definitions, With the Addition of Sea Surface Temperature (SST) and Salinity (SSS))

Field	E2.1‐G (f3)	E2.1‐G (f2)	E2.1‐G (f1)	E2.1‐H (f2)	E2‐R	E2‐H
OTR	**0.68**	**0.68**	**0.68**	0.67	0.66	0.63
ASR	0.84	0.84	0.84	**0.85**	0.79	0.78
MSU‐TMT	0.88	0.89	0.88	**0.90**	**0.90**	**0.90**
MSU‐TLS	0.69	0.64	0.69	0.62	**0.73**	0.71
TOTAL CLOUD	**0.33**	0.32	0.32	0.31	0.19	0.17
LOW CLOUD	**0.36**	0.35	0.35	0.34	0.16	0.12
SLP (DJF)	0.75	0.76	0.75	**0.81**	0.78	0.71
SLP (JJA)	0.82	0.82	0.82	**0.83**	0.79	0.75
SAT (DJF)	**0.90**	**0.90**	**0.90**	0.89	**0.90**	0.88
SAT (JJA)	0.89	**0.90**	**0.90**	**0.90**	**0.90**	0.87
PRECIP	0.51	**0.52**	0.51	0.51	0.50	0.45
EWSS	**0.81**	**0.81**	**0.81**	0.77	0.78	0.71
SST	0.90	0.90	0.90	0.90	**0.91**	0.86
SSS	0.72	**0.73**	**0.73**	0.57	0.63	0.54

*Note*. The highest scores across the coupled models for each field are highlighted in bold. Note that for the E2 models, the output data are from 1979–2004, while the target climatologies are as described above.

Any overall ranking of performance is by necessity ad hoc given the subjective choice of metrics and weighting, and not determinative of every metric, but across a range of measures, the E2.1‐G (f2,f3) are the best performing configurations considered here. There are small degradations of skill for the MSU diagnostics (though not for the trends Miller et al., 2020). E2.1‐H has slightly better SLP patterns, but the differences in atmospheric variables are minor, especially compared to the improvements of all E2.1 configurations with respect to E2.

## 6 Climate Sensitivities

As part of the DECK simulations requested by CMIP6, we performed a number of idealized simulations (1pct4xCO2, abrupt4 × CO_2_) as well as some related simulations (abrupt2 × CO_2_ with the coupled and q‐flux ocean versions) (all performed with the f1 background composition). The summary of various metrics of climate sensitivity (along with the comparison to the previous models) is seen in Table 6. We note that the effective climate sensitivity as calculated by the Gregory method (Gregory et al., 2004) almost always underestimates the true long‐term ECS by 10% to 20% (Dunne et al., 2020). The perhaps more relevant TCR is slightly larger in the E2.1 models than previously, consistent with a smaller rate of mixing of heat into the ocean (and slightly smaller present‐day overall radiative imbalance (Table 2).

**Table 6:** Climate Sensitivities to 2 × CO_2_ (°C) Estimated Multiple Ways (Note That Not All Calculations Have Been Completed With All Versions)

Model version			ECS		
and configuration	ECS_qflux_	CS_Eff_	from 4 × CO_2_	from 2 × CO_2_	TCR
E2.1‐G (NINT)	3.0	2.7	3.2	3.6	1.8
E2.1‐H (NINT)	"	3.1	3.5	3.4	1.9
E2.1‐G (OMA)	2.9	2.6			1.6
E2.1‐H (OMA)	"	3.1			2.0
E2.1‐G (MATRIX)	2.9	2.8			1.8
E2.1‐H (MATRIX)	"				2.0
E2.1‐G (TOMAS)	3.1				
E2‐R (NINT)	2.7	2.1	2.3	2.6	1.4
E2‐H (NINT)	"	2.3	2.5		1.7
E2‐R (TCADI/OMA)	3.0	2.4			1.6
E2‐H (TCADI/OMA)	"	2.5			1.8

*Note*. Equilibrium Climate Sensitivity (ECS) is defined from multi‐millennial coupled simulations, or from a q‐flux (slab ocean) model (ECS_qflux_). CS_Eff_ is from a linear extrapolation of years 1–150 results in the abrupt4 × CO_2_ simulations (Gregory et al., 2004). Transient Climate Responses (TCRs) are taken from year 70 in the 1pct4 × CO_2_ simulation.

The relative stability of the climate sensitivity from E2 to E2.1 is however due to two counteracting influences. First, as discussed in Miller et al. (2020), the effective radiative forcing associated with a doubling of CO_2_ is 15% smaller (3.59 compared to 4.19 W m^−2^) in the E2.1 model than it was in E2 and closer to the canonical 3.7 W m^−2^ (Myhre et al., 2013). This is consistent with higher water vapor content and greater LW cloud forcing which reduce the baseline contribution of CO_2_ to longwave opacity, and hence reduce the sensitivity to opacity changes. Second, the changes to cloud feedbacks associated with the increase in supercooled cloud water make the overall cloud feedbacks more positive (by reducing the negative cloud phase feedback (Tan et al., 2016; Zelinka et al., 2020). Thus, the impact to 2 × CO_2_ is only slightly changed, though the normalized sensitivity has increased substantially from 0.62°C W^−1^ m^2^ to 1.00°C W^−1^ m^2^ (using the ECS from 2 × CO_2_), or similarly from 0.58 to 0.87 W^−1^ m^2^ (using the long‐term response to 4 × CO_2_).

## 7 Conclusions

As computational resources increase, the temptation at many climate modeling centers is to increase resolution (and therefore compute time) such that the overall throughput of the model stays roughly constant. In contrast to that strategy, the increment from the E2 to E2.1 versions focused instead on fixes, better calibrations and in a few cases, improved parameterizations. This was embarked on in parallel with a far more extensive upgrade for the E3 code (including, new topologies, new dynamical cores, higher horizontal and vertical resolution, and new moist physics), which will be reported elsewhere. The question then arises, as to whether the strategy used for E2.1 can provide a worthwhile increase in skill with negligible costs of additional runtime, more efficiently than the E3 strategy. The answer to that is a definitive yes.

Skill scores in E2.1 are consistently (though not universally) higher in fields that were specifically tuned for as well as in emergent properties (such as the PDO patterns) that were not. Improvements are physically coherent across fields, particularly in the Southern Ocean where the positive changes have been seen in the ocean, atmosphere and cryosphere. Indeed, these are the first GISS models to have a credible simulation of the Southern Oceans.

Nonetheless, we note the limitations of this approach and the stubborn persistence of long‐term biases. Notably, while many cloud properties improved, the lack of sufficient marine stratus is still apparent. Similarly, the persistence of a double‐ITCZ, and excessive hemispheric symmetry in the zonal mean tropical diagnostics has not been ameliorated to any significant extent. These features have however been almost eliminated in the preliminary E3 simulations which have had the benefit of higher resolution, greatly improved moist physics and more comprehensive calibration (Cesana et al., 2019). It is also apparent that minor retunings are not able to compensate for a model top that is too low for a realistic stratospheric circulation or quasi‐biennial oscillation (Orbe et al., 2020; Rind et al., 2014).

Within the broader constellation of the multimodel ensembles used in CMIP, true structural diversity continues to be a necessary component for any multimodel projection to have a hope of spanning the “truth” (Knutti et al., 2013). Better‐calibrated lower resolution models and more sophisticated higher resolution models here can play a significant role in expanding that diversity and avoiding the potential danger of similar, and perhaps problematic, new assumptions being adopted by all model groups as they jointly improve such features as cloud and aerosol microphysics (Andrews et al., 2019; Gettelman et al., 2019; Golaz et al., 2019). The apparent increase in climate sensitivity to doubled CO_2_ in some of the next‐generation models (Dunne et al., 2020; Forster et al., 2019; Zelinka et al., 2020) whether realistic or not, is very concerning. If this is a reflection of the real world, climate impacts are likely to be greater than we have up to now anticipated, and if it is not, then it raises serious questions about model independence and underlines the importance of true structural diversity. We simply note that the model sensitivity seen in the E2.1 models (∼3°C) is near the center of the traditionally assessed range of 1.5°C to 4.5°C. While our understanding of the uncertainty in climate sensitivity has improved enormously since the Charney report (Charney et al., 1979), the latest assessments do not fundamentally challenge it (Sherwood et al., 2020).

## 8 Data and Code Availability

All standard data from the piControl, historical, abrupt4 × CO_2_, and 1pctCO_2_ simulations discussed here are publicly available in the CMIP6 archive through multiple nodes of the Earth System Grid Federation (Table  7). The code used corresponds to the E2.1 tag in the ModelE git repository available from the NCCS CDS system. Additional selected diagnostics from the 2 × CO_2_ runs and q‐flux versions (mentioned in Table  6), and further derived data from the simulations (including the diagnosed MSU and SSU fields) are available online (https://portal.nccs.nasa.gov/GISS_modelE/E2.1/).

**Table 7:** Model Experiments in CMIP6, Simulation Identifiers (Using Standard Regular Expression Format), and DOIs for the Ensemble

Model version	Experiment	ripf number	DOI
E2.1‐G	piControl	r1p[1345]f1	10.22033/ESGF/CMIP6.7380
	historical	r[1‐10]p[1345]f[123]	10.22033/ESGF/CMIP6.7127
	abrupt4 × CO_2_	r1p[13]f1	10.22033/ESGF/CMIP6.6976
	1pctCO_2_	r1p[13]f1	10.22033/ESGF/CMIP6.6950
E2.1‐H	piControl	r1p[1345]f1	10.22033/ESGF/CMIP6.7381
	historical	r[1‐5]p[13]f[12]	10.22033/ESGF/CMIP6.7128
	abrupt4 × CO_2_	r1p[13]f1	10.22033/ESGF/CMIP6.6977
	1pctCO_2_	r1p[13]f1	10.22033/ESGF/CMIP6.6951

## Data Availability

The water vapor and precipitation data sets used in this work were obtained from the obs4MIPs (https://esgf-node.llnl.gov/projects/obs4mips) project hosted on the Earth System Grid Federation (https://esgf.llnl.gov). MSU data are produced by Remote Sensing Systems and sponsored by the NOAA Climate and Global Change Program and are available online (www.remss.com). SSU data are from NOAA / NESDIS Center for Satellite Applications and Research. Ocean Heat Content data were taken from NOAA NCEI (https://www.nodc.noaa.gov/OC5/3M_HEAT_CONTENT/).
